# Enhancing therapeutic efficacy: sustained delivery of 5-fluorouracil (5-FU) via thiolated chitosan nanoparticles targeting CD44 in triple-negative breast cancer

**DOI:** 10.1038/s41598-024-55900-1

**Published:** 2024-05-19

**Authors:** Sadia Anjum, Faiza Naseer, Tahir Ahmad, Faryal Jahan, Halima Qadir, Rabia Gul, Kousain Kousar, Atif Sarwar, Abdallah Shabbir

**Affiliations:** 1 Department of Biology, University of Hail, Hail, Saudi Arabia; 2 Department of Biosciences, Shifa Tameer e Millat University, Islamabad, Pakistan; 3 Industrial Biotechnology, Atta-ur-Rahman School of Applied Biosciences, National University of Sciences and Technology, Islamabad, Pakistan; 4 Shifa College of Pharmaceutical Sciences, Shifa Tameer e Millat University, Islamabad, Pakistan

**Keywords:** CD44, 5-Fluorouracil, Hyaluronic acid, Thiolated chitosan, Triple-negative breast cancer, Nano drug delivery system, Cancer, Nanoscience and technology

## Abstract

Our current study reports the successful synthesis of thiolated chitosan-based nanoparticles for targeted drug delivery of 5-Fluorouracil. This process was achieved through the ionic gelation technique, aiming to improve the efficacy of the chemotherapeutic moiety by modifying the surface of the nanoparticles (NPs) with a ligand. We coated these NPs with hyaluronic acid (HA) to actively target the CD44 receptor, which is frequently overexpressed in various solid malignancies, including breast cancer. XRD, FTIR, SEM, and TEM were used for the physicochemical analysis of the NPs. These 5-Fluorouracil (5-FU) loaded NPs were evaluated on MDA-MB-231 (a triple-negative breast cell line) and MCF-10A (normal epithelial breast cells) to determine their in vitro efficacy. The developed 5-FU-loaded NPs exhibited a particle size within a favorable range (< 300 nm). The positive zeta potential of these nanoparticles facilitated their uptake by negatively charged cancer cells. Moreover, they demonstrated robust stability and achieved high encapsulation efficiency. These nanoparticles exhibited significant cytotoxicity compared to the crude drug (*p* < 0.05) and displayed a promising release pattern consistent with the basic diffusion model. These traits improve the pharmacokinetic profile, efficacy, and ability to precisely target these nanoparticles, offering a potentially successful anticancer treatment for breast cancer. However, additional in vivo assessments of these formulations are obligatory to confirm these findings.

## Introduction

Triple-negative breast cancer ranks among the most aggressive types of breast cancer, and its name is attributed to the lack of three major receptors conventionally targeted for breast cancer treatment. These include receptors for HER2 (epidermal growth factor), estrogen and progesterone. The exact initiating factor behind triple-negative breast cancer is unidentified^1^. The etiological causes include age, genetic tendency, hormonal changes and age (> 40). Lifestyle factors like obesity, consumption of alcohol, radiation exposure, and reproductive factors also play a substantial role. Cancer can be categorized based on the involvement of germ cells or somatic cells^2^. Germ cells pass genetic information from one generation to the next. On the other hand, somatic cells are all the other cells in the body; mutations in these cells can lead to cancer. In breast cancer, the most common type involves somatic mutations, which occur in the somatic cells of the breast tissue^3^.

These mutations are acquired during a person's lifetime and are not inherited. They are often the result of various environmental and lifestyle factors, such as exposure to radiation, hormonal influences, or other carcinogens. However, some breast cancer cases can be attributed to inherited genetic mutations in germ cells^4^. These mutations are passed down from parents to their children. The two most well-known genes associated with inherited breast cancer are BRCA1 and BRCA2. When these genes carry mutations, they significantly enhance the risk of mounting breast cancer and other gynaecological malignancies, such as ovarian cancer. Inheriting a mutated copy of BRCA1 or BRCA2 substantially raises the risk compared to the general population. Apart from BRCA1 and BRCA2, other less common genes can also be involved in hereditary breast cancer. Genetic testing can help identify individuals with these mutations, enabling them to take proactive measures to reduce their risk or undergo regular screenings for early detection^5^.

Preventive measures involve maintaining a healthy lifestyle, including a nutritious diet, avoiding alcohol, and engaging in gentle exercise with medical advice^2^. Regular check-ups and screenings are essential. If any breast changes are noticed, it's crucial to consult a doctor promptly, regardless of recent normal mammogram results^6^. Treatment options depend on the type and stage of the cancer. Medication options such as chemotherapy and/or hormone therapy may be used. It can be required to undergo surgical procedures such as mastectomy or lumpectomy along with potential breast reconstruction. High-risk women may consider preventive surgery. Radiation therapy can also destroy cancer cells^7^.

While these procedures aim to remove cancerous tissue, they may lead to changes in breast appearance and cause emotional distress for the patient. Additionally, radiation therapy, which uses high-intensity X-rays to eradicate cancer cells, can lead to skin irritation, redness, and fatigue^8^. Chemotherapy, involving drugs to destroy or inhibit cancer cells, often brings about side effects such as nausea, hair loss, fatigue, and toxicity. Hormone therapy works by blocking or lowering hormone levels, but it can lead to menopausal symptoms and may have long-term effects^9^. Immunotherapy, designed to help the immune system attack cancer cells, may sometimes have a limited response^10^. Patients and healthcare providers must carefully weigh these treatment options and consider their drawbacks when deciding on the most suitable course of action^11^.

Nanoparticles hold promise in breast cancer treatment, offering targeted drug delivery, improved drug solubility, prolonged drug release, and decreased side effects^12^. They can overcome drug resistance and aid in early detection through imaging. It's important to note that using nanoparticles for breast cancer treatment is an active area for clinical investigation. In contrast, the mentioned treatments (radiation therapy, surgery, hormone therapy and chemotherapy) are well-established and are widely used for breast cancer management. Nanoparticles offer several potential advantages that make them an area of interest in cancer therapeutics^13^.

Current nanomaterial-mediated drug delivery for breast cancer encompasses a diverse array of strategies aimed at enhancing the efficacy and specificity of anticancer therapeutics while mitigating off-target effects and systemic toxicity. Nanoparticles, such as liposomes, polymeric nanoparticles, dendrimers, and inorganic nanoparticles like gold or iron oxide, are commonly employed as carriers for chemotherapeutic agents, targeted therapies, or nucleic acid-based drugs^14^. These nanomaterials offer several advantages, including prolonged circulation time, selective accumulation in tumor tissues via the enhanced permeability and retention (EPR) effect, and the ability to encapsulate hydrophobic drugs. The functionalization of nanomaterials with targeting ligands, such as antibodies or peptides, further enhances their tumor specificity by facilitating receptor-mediated endocytosis^15^ Moreover, stimuli-responsive nanocarriers can release their payload in response to specific triggers in the tumor microenvironment, thereby improving drug bioavailability and therapeutic efficacy. Despite these advancements, challenges such as limited drug loading capacity, premature drug release, and potential immunogenicity remain areas of active research and development in nanomaterial-mediated drug delivery for breast cancer^7^.

The cytotoxic drug 5-fluorouracil (5-FU) is the major treatment choice against TNBC. It is usually given as an injection into a vein or as a topical cream. It is a part of the class of drugs known as anti-metabolites, which interfere with the synthesis and function of DNA and RNA in rapidly dividing cancer cells, leading to their destruction^16^. In breast cancer treatment, 5-FU is often combined with other chemotherapy drugs to maximize its effectiveness^17^. It may be given before surgery as a neoadjuvant therapy to reduce the tumor burden, or it can be administered as a post-surgical treatment to target any residual malignancy and lessen the menace of recurrence^18^.

Chitosan (Cs) is a naturally occurring biocompatible and biodegradable polysaccharide. Chitosan thiolation (TCs) decreases cytotoxicity and enhances mucoadhesive properties and stability. This polymer's versatile, functional group possibilities are ideal for drug loading and surface functionalization for targeted delivery^19^. The availability of active thiol (-SH) groups on TCs and HA allow them to attach with binding pockets on receptors present on the surface of mucous membranes through ionic and covalent electrostatic bonding^20^. Chitosan, a linear compound with a positive charge, is similar to naturally occurring glycosaminoglycan and can be modified chemically and physically due to its active amine group^21^. The biological characteristics of the chitosan can be enhanced through ionic gelation, a method involving the establishment of covalent or ionic bonds with negatively charged molecules from other polymers^22^. Hyaluronic acid (HA), a hydrophilic mucopolysaccharide with a negatively charged structure, is naturally present in the junctions, synovial fluid, connective tissues and extracellular matrix of joints^23^. Its functional reactive sites make it ideal for therapeutic applications, and it binds to CD44 receptors, which are overexpressed in solid tumors. This receptor-specific binding allows hyaluronic acid to be utilized in drug delivery systems, targeting tumor cells while minimizing its penetration into normal tissues^24^.

**Figure 1:**
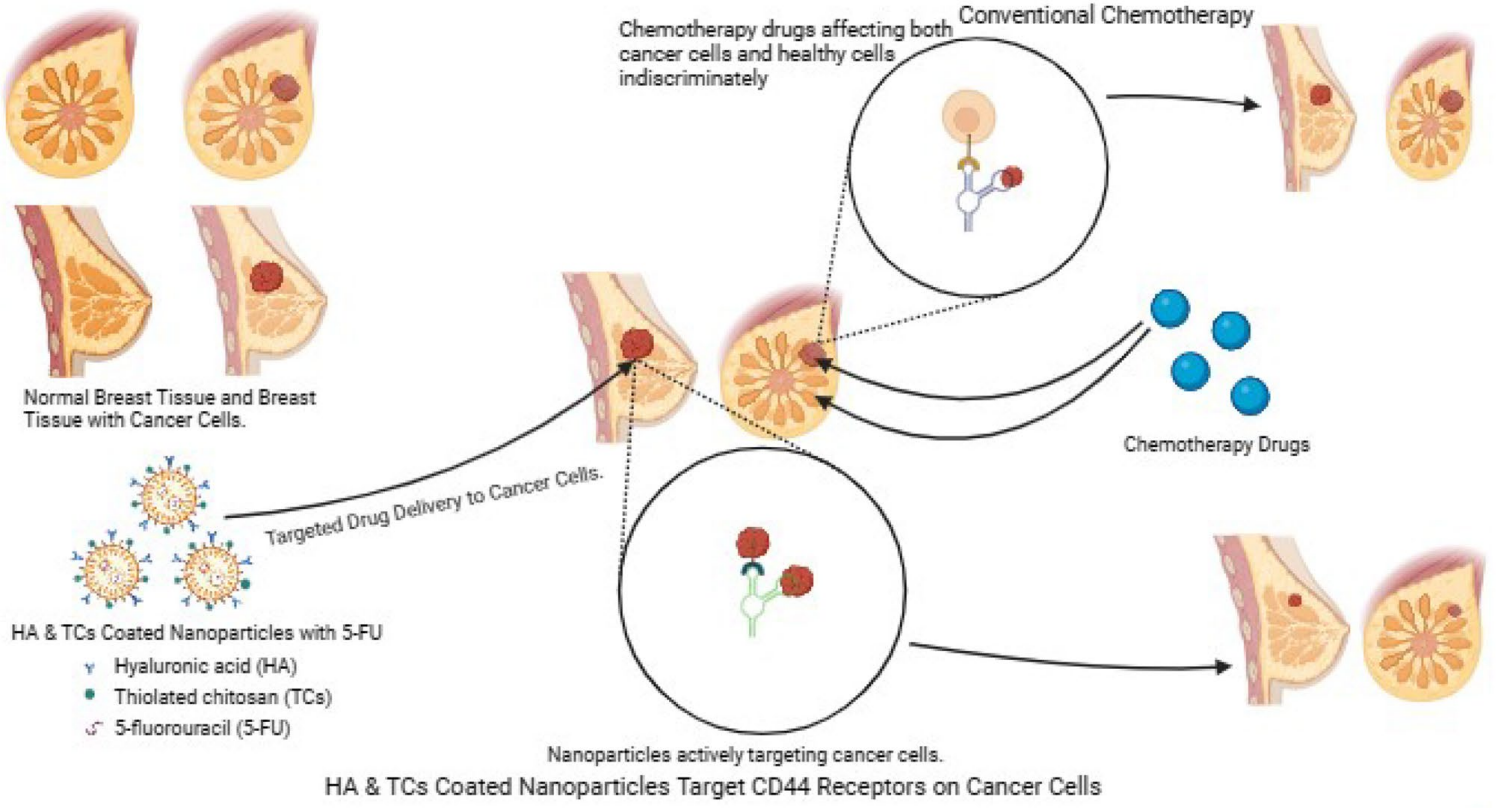
Schematic illustration of targeted delivery of HA-coated 5-FU in ThCs-NPs formulation for the treatment of breast cancer compared to concentional chemotherapeutic drugs.

This study used 5-FU, a potent anticancer therapeutic moiety, to formulate a drug delivery system (DDS) using HA-coated TCs. The primary objective was to target tumor sites by binding to CD44 receptors and releasing therapeutic moiety through receptor-mediated endocytosis at the specific cells^23,24^ Fig. 1. This approach aims to reduce toxicities associated with cytotoxic drugs, as HA exhibits minimal penetration into normal tissues. By encapsulating 5-FU within the nanoparticles, we enhanced its delivery to tumor cells, potentially improving the efficacy of the treatment while minimizing adverse effects on healthy tissues^25^.

## Materials and methods

### Materials

Chitosan (Cs) having low molecular weight (50–190 kDa) with 80% deacetylation, Acetic acid, Thioglycolic acid (TGA), Sodium hydroxide (NaOH), Tripolyphosphate polyanions (TPP), Hyaluronic acid having high molecular weight of 1500 kDa, Dialysis membrane (12,000–14,000 Mw cut-off retention capacity) and Sodium borohydride were acquired from Sigma-Aldrich (Germany) through worldwide scientific store. The reagents Hydroxylamine, artificial mucin, Glacial acetic acid, 1-ethyl-3-(3-dimethylaminopropyl) carbodiimide hydrochloride (EDC), Ellman's reagent (5, 5'-dithiobis (2-nitrobenzoic acid; DTNB) and NHS (N-hydroxysuccinimide) and Calcium chloride (CaCl_2_) were acquired from Merck (Germany) and supplied by Adnan Traders (Pakistan). Distilled water (analytical grade) was supplied by the Industrial Biotechnology Lab, National University of Science and Technology (NUST), Islamabad, Pakistan.

### In silico analysis

#### Retrieval and preparation of the target macromolecule and ligand

The crystallographic structure of the targeted CD44 protein was retrieved from PDB (Protein Databank) with PDB id: 1UUH and downloaded in PDB format. Protein was prepared for docking in Discovery Studio Visualizer by removing attached ligands and water molecules to avoid any unwanted interaction during the docking process. Polar hydrogen atoms were added to the target protein^26^. The structure of the ligand was drawn using a chem sketch. Subsequently, the prepared protein and saved ligand were uploaded to the virtual docking software Pyrx. Open babel was used to minimize the energy of ligand under the universal force field and converted to pdbqt before docking.

#### Molecular docking of ligand (HA) and receptor (CD44)

The selected ligand and desired target were docked using Autodock vina 4.2 suits of PyRx. The number of runs was set to 200 for each docking. A grid map was resized to cover the active sites of the target protein and had an exhaustiveness of 8, while other docking parameters were set as default^27^. The results obtained after virtual docking were documented as binding affinity (kcal/mol). The Discovery Studio visualizer visualized the 2D and 3D interactions between ligands and macromolecules. The number of hydrogen bonds formed by the ligand with residues of macromolecule was documented.

#### Thiolated chitosan solution preparation

To make a solution of thiolated chitosan (ThCs), 1% acetic acid solution was used to make 1% chitosan solution, with 50 mM of EDC and 7.0 ml of TGA to the chitosan solution. The amide bond formation between chitosan and thioglycolic acid starts by activating the -COOH group on TGA^23^. 1% hydroxylamine was added to this solution to avoid the oxidation phenomena. 1 M NaOH was mixed to adjust the pH of the mixture to 5.8. With constant stirring, the resulting mixture was passed through the dialysis membrane for three consecutive days to remove any unbound TGA. The dialysis medium of 5 mM HCl (5L solution) was freshly prepared 4 times daily. The dialyzed solution was stored at -80 °C and then lyophilized for further storage. The final product obtained after lyophilization was a white amorphous material stored at 4 °C until the experiment^24^.

#### Ellman's assay for thiol group quantification:

A spectroscopy-based study was conducted to evaluate the extent of the substitution of the –SH group of ThCs. For this purpose, 5 mg ThCs and 0.25 ml of phosphate buffer saline (PBS) were dissolved in distilled water to maintain the pH-7. Then, 0.5 ml of Ellman's reagent was added, and the mixture was incubated for 2 h at 25 °C. Afterwards, the mixture was centrifuged for 10 min at 23,700 rpm to collect supernatant. The spectrophotometer (at 420 nm) was used to measure the absorbance of the collected supernatant^27^. The control samples with non-thiolated chitosan were used for comparison. The density of the thiol group of thiolated chitosan was calculated by comparing the results with the corresponding control samples. A standard curve for TGA was also built to validate the analysis^28^.

#### Formulation of Hyaluronic acid functionalized thiolated chitosan nanoparticles

Hyaluronic Acid (HA)-coated ThCs-NPs were synthesized using standard laboratory apparatus, including a beaker, magnetic stirrer, and dropper. The synthesis process relied on the principle of ionic crosslinking, where the electrostatic attraction between the NH_2_ (from ThCs) and the COOH group (from HA) led to the creation of nanoparticles. A solution of ThCs was prepared, and 0.1 mg/ml TPP was gradually added as a polylinker. This step facilitated the formulation of Empty ThCs-NPs as a starting point. Subsequently, the HA solution was incorporated into the mixture, allowing it to attach to the surface of the ThCs-NPs. This integration resulted in the successful formation of HA-coated ThCs-NPs^29^.

A slight modification in the above procedure also prepared 5-FU loaded NPs. 0.15 mg/ml of 5-FU and 0.10 ml of TPP were poured dropwise through an injection at 560 rpm for 10 min, resulting in 5-FU-Loaded NPs^23^. To ensure uniform dispersion and homogenization of all nanoparticle formulations, they were sonicated at 30 mA for 15 min. Following probe sonication, the drug-loaded nanoparticles were treated with HA for functionalization, followed by centrifugation, lyophilization, and storage at 4 °C until further use^24^.

#### Optimization of formulation parameters for 5-FU-Loaded-NPs

The Box-Behnken factorial design (BBD) is a software for methodical optimization. We employed BBD to synthesize a precise drug delivery system in nanoforms quickly. This research optimized HA-coated 5-FU in ThCs-NPs using the Design of Expert (DOE) Software version 8.0.7^30^. The concentrations of TCs, 5-FU, and HA were the independent variables considered for optimization, while the size of nanoparticles, zeta potential and polydispersity index (PDI) were the dependent variables. This approach selected the best-optimized nanoformulation, and further characterization was conducted to evaluate different physicochemical parameters (Table 1)^31^.

**Table 1:** The Box-Behnken design depicted nanoparticle size, the PDI and the zeta potential for different concentrations of TCs, 5-FU and HA.

Run	HA mg	TC mg	5-FU mg	Particle size nm	PDI	Zeta potential + /-mV
1	50.00	60.00	0.10	1444	1	− 9.84
2	50.00	20.00	0.55	752.8	1	10
3	37.50	100.00	0.10	460.7	0.49	25.6
4	37.50	100.00	1.00	587.2	0.591	− 23.7
**5**	**25.00**	**60.00**	**0.10**	**327**	**1**	**8.96**
6	50.00	100.00	0.55	436	0.512	− 21.6
7	50.00	60.00	1.00	1683	1	7.5
8	25.00	20.00	0.55	396.2	0.524	− 27.6
9	25.00	100.00	0.55	416.7	0.584	12
10	37.50	20.00	0.10	3191	1	10
11	25.00	60.00	1.00	438	0.678	12
12	37.50	20.00	1.00	592.5	0.433	38.9

The highlighted values are the ones selected for proceeding with NP synthesis.

#### Physicochemical characterization of HA-coated 5-FU in ThCs-NPs

Characterization plays a crucial role in assessing the capability of the nanoparticle formulation for drug delivery and uptake at targeted areas in the cells. SEM, TEM, Zetasizer, X-ray Diffraction, FTIR spectrometer and Raman Spectroscopy were employed.

#### Scanning electron microscopic (SEM) analysis for HA-coated 5-FU in ThCs-NPs

The surface chemistry and precise microstructure of Empty HA-ThCs and HA-coated 5-FU in ThCs-NPs were observed through SEM using MIRA3 (TESCAN, Czech Republic). Using lyophilized powder of nanoparticles by fixing on aluminium stubs with gold coating, images were captured at a 15 kV voltage using accelerated electrons^32^.

#### Transmission electron microscopic (TEM) analysis for HA-coated 5-FU in ThCs-NPs

To examine nanoparticles to confirm drug loading and determine morphology (shape and size), TEM with specifications of JEOL/JEM 2100, Akishima, Tokyo, Japan, was used. A single drop of Empty HA-ThCs and HA-coated 5-FU in ThCs-NPs were lyophilized on a copper grid, and the images were captured at 200 kV^23^.

#### Zeta analysis for HA-coated 5-FU in ThCs-NPs

The zeta analysis of Empty HA-ThCs and HA-coated 5-FU in ThCs-NPs was conducted using the Malvern Zetasizer Nanos ZS90 (UK) to calculate the average values for nanoparticle size, zeta potential, and PDI. The Zetasizer provided hydrodynamic diameter values of the diluted nanoparticles (1:10). Five batches were evaluated on the Zetasizer to get an average value and standard deviation calculation. The PDI of the nanoparticles represents the ratio of molecular weight (Mw) to number-average molecular mass (Mn)^33^.

$$PDI = \frac{{M_{w} }}{{M_{n} }}$$

#### Raman spectroscopic analysis for HA-coated 5-FU in ThCs-NPs

The Raman Spectroscopic instrument used by Thermo Fisher Co., Ltd. (United States) was used to study the rotational and vibrational modes of molecules in empty and 5-FU nanoparticles in magnetic fields. The Raman shift was detected with a laser power of 150 mW and at a wavelength range of 0–3500 cm^−1^ with an excitation wavelength of 780 nm. The observations were collected in pectoral form with the representation of spectral data.

#### X-ray diffraction (XRD) analysis for HA-coated 5-FU in ThCs-NPs

XRD analysis of empty and HA-coated 5-FU in ThCs-NPs nanoparticles was conducted using an X-ray diffractometer D8 ADVANCE (Bruker, Germany) at 25 °C. The analysis aimed to assess the crystalline nature of nanoparticles. The results were observed in the form of an XRD pattern at an angular range of 10°–50° with 2θ in a continuous mode (step size: 0.02, 2θ and step time: 1 min).

#### Fourier transform infrared spectroscopy (FTIR) for HA-coated 5-FU in ThCs-NPs

Identification of functional groups was conducted on an FTIR spectrometer (Perkin Elmer, MA, United States) and confirmed the effective encapsulation of 5-FU inside the ThCs nanoparticles^34^. The characteristic points of lyophilized active compounds of Empty and drug-loaded nanoparticles were identified.

#### Drug loading (DL) and efficiency of encapsulation (EE)

Measuring drug content in the solution surrounding the nanoparticles indirectly reflects the amount of drug incorporated within the nanoparticles. This method was employed to quantify the amount of 5-FU loaded into the nanoparticles by ionic gelation method and to determine the encapsulation efficiency of these formulations^35^. The nanoparticle formulation was centrifuged at 13,700 rpm for 1 h to perform this analysis. The resulting supernatant was collected. The syringe filter was used for filtration, and the filtrate was analyzed for the concentration of unbound 5-FU by UV–Vis spectrophotometer (NanoDrop 2000c; Thermo Scientific, Wilmington, US) at 266 nm by following formulas^36^.

$$\% Drug\, loading\, \left( {DL} \right) = \frac{{\left( {W_{1} - W_{2} } \right)}}{{W_{3} }} \times 100$$

W1 = Total concentration of 5-FU used, sW2 = amount of 5-FU present in supernatant,

W3 = Quantity of ThCs polymer (constituting the nanoparticles).

$$\% Encapsulation \,efficiency\, \left( {EE} \right) = \frac{{\left( {W_{1} - W_{2} } \right) }}{{W_{1} }} \times 100$$

W1 = Overall quantity of 5-FU, and W2 = Unbound 5-FU detected in the supernatant.

#### Calibration plot for 5-FU in distilled water and phosphate buffer at pH 7.4

This procedure aimed to establish a calibration curve for 5-FU. Following the guidelines outlined in the British Pharmacopoeia (BP), 5-FU concentrations ranging from 0.1 to 1 mg/mL were prepared by diluting the drug in distilled water (served as a reference). The prepared samples were investigated using a UV-Vis spectrophotometer at 266 nm. Subsequently, a calibration curve was constructed utilizing MS-Excel, which played a pivotal role in determining unknown concentrations of 5-FU within samples of nanoparticle formulation^37^.

A calibration curve based on drug concentration was constructed to measure the quantity of 5-FU in the release medium. Initially, 1 mg 5-FU was dissolved in 10 ml PBS (served as reference) at pH-7.4. The prepared solution was sonicated for 8 to 10 minutes to ensure thorough suspension. Then, the serial dilutions from this solution were prepared to plot the calibration curve using GraphPad Prism 9 software. The drug dilutions were analyzed at a wavelength of 266 nm using a UV spectrophotometer. The data was used to calculate the in vitro 5-FU release profile^38^.

#### In vitro analysis: release of 5-FU from nanoparticles

A dialysis membrane assay was performed using a dialysis bag immersed in PBS with pH 7.4 (normal cells) and 6.8 (cancer cells), maintained at 37 °C. A lyophilized 5 mg of HA-coated 5-FU in ThCs-NPs powder was introduced into a dialysis bag containing 50 ml PBS at both pH, with continuous stirring at 1000 RPM on a magnetic hot plate. At predefined time points, including start, 1, 2, 3, 4, 7, 12, 24, 48, and 72 h, 1.5 ml of the sample from the solution was collected and subjected to analysis at 266 nm by UV spectrophotometer. A standard calibration curve of 5-FU was plotted on an MS Excel sheet, employing observations derived from the following formula. The experiment was performed in triplicate, ensuring robust and reliable outcomes^23,37^.

$$percentage\, of\, drug\, released\, in - vitro = \frac{the\, amount\, of\, 5 - FU\, drug\, released\, in\, the\, buffer}{{the\, total\, amount\, of\, 5 - FU\, drug\, initially\, added}} \times 100$$

#### Drug release kinetics

Various kinetic methods such as Zero-order release kinetics, First-order release kinetics, Higuchi Equation (Diffusion method), Erosion Model, and Korsmeyer-Peppas Kinetic Model were utilized for *in-vitro* release from nanoparticles.

#### Zero-order release kinetics

Following zero-order kinetics, the pace of drug release remains steady and continuous from the matrix of the drug delivery system^39^. The gradient of this graphical representation unveils the zero-order release constant by the following formula:

$$W = k_{1} t$$

where: W = Total release of drug, k_1_ = drug release constant for zero order, t = time (hours).

#### First-order release kinetics

In pharmacokinetics, the way 5-FU is released from its nanocarrier follows the principles of first-order kinetics, a fascinating and clinically relevant concept. Unlike the steady pace of zero-order kinetics, this process varies with the drug concentration^40^. A graph elegantly illustrates this, depicting the logarithmic transformation of cumulative drug release over time. The following formula precisely describes this important aspect:

$$\ln \left( {100 - W} \right) = \ln 100 - k_{2}$$

where: W = Total release of drug, k_2_ = drug release constant for first order.

#### Higuchi equation (diffusion method)

The Higuchi model elucidates the non-erodible diffusion-based therapeutic moiety release from the drug matrix^41^. The cumulative release of 5-FU was plotted and compared to the square root of time to unveil its diffusion behavior by the following formula:

$$W = k_{4} t$$

where: W = Total drug concentration released in time, k_4_ = Higuchi dissolution rate constant.

#### Erosion model: Hixon Crowell's cube root equation

The Hixon-Crowell model elucidates the liberation of the therapeutic moiety from the drug matrix through dissolution triggered by altering the diameter and surface area of the drug nanoparticles. This model portrays the drug release process as a sequence beginning with erosion, followed by diffusion^40^. The erosion model is mathematically given below:

$$\left( {100} \right)^{1/3} 100^{1/3} - W = - k_{3} t$$

where: k_3_ = Hixon release constant, W = Total drug concentration released due to dissolution.

#### Korsmeyer-Peppas kinetic model

This model intricately elucidates the process of drug liberation from polymeric systems. It considers multiple release mechanisms, encompassing water diffusion within the nanoparticle, swelling, and dissolution from the drug matrix^41^. The model characterizes the release of drugs over time in an exponential manner, as expressed by the following formula:

$$Mt/M\infty = k5tn$$

Here, Mt/M∞ signifies the percentage of drug release at time t; n denotes the diffusion exponent governing drug release; k5 represents a constant that encapsulates geometrical attributes of the sustained drug delivery system. For instance, *n* = 0.45 indicates Fickian diffusion, which holds crucial insights into the mechanism of drug release.

#### In vitro anticancer activity of NPs

The cytotoxicity of 5-FU encapsulated in HA-ThCs nanoparticles was investigated using breast cancer cell lines. Specifically, we focused on assessing the nanoparticles' effect on TNBC's viability and compared this with the effects of commercially available 5-FU administered at equivalent concentrations. The breast cancer cell lines utilized in this study were obtained from Dr. Tahir Mehmood, Associate Professor at the University of Veterinary and Animal Sciences (UVAS), Lahore, Pakistan. These cell lines were initially sourced from the National Institute of Health (NIH), Islamabad, Pakistan.

Cell lines were seeded in RPMI 1640 media with 10% FBS (Gibco) and 1% Penstrep comprising 100 units of penicillin and 100 µg of streptomycin (Gibco). All cell lines were maintained in a controlled environment at room temperature with 5% CO_2_ and 95% humidified air. Upon reaching the exponential growth phase, the cells were subjected to experimentation and cultured in T75 (75 cm2) tissue culture flasks to ensure optimal conditions for the cytotoxicity assessment of breast cancer^42^. The following are the groups for in vitro experiments:

Group 1: MDA-MB-231(Control), Group 2 and 3: MDA-MB-231 cells treated with crude 5-FU and 5-FU-NPs. Group 4: MCF-10A cells (Control), Group 5 and 6: MCF-10A cells treated with crude 5-FU and 5-FU-NPs.

#### Morphological analysis

Distinctive apoptotic characteristics include membrane cellular shrinkage, blebbing, cellular rounding and nuclear and cytoplasmic condensation. To document these alterations, normal breast cells (MCF-10A) and triple-negative breast cancer cells (MDA-MB-231) were cultured in a 96-well plate with a concentration of 1 × 10^6^ cells per well (100 µl). The next day, the cells were exposed to various concentrations, including 10, 50 and 90 μg/ml of crude 5-FU compared to 5-FU-NPs dissolved in PBS. The change in physiological appearance induced by the pure drug and the nanoparticles was observed at 12 and 24 h using an optical inverted microscope (phase-contrast) at 40X^23^.

#### Cell viability analysis using trypan blue exclusion assay

The assay was employed to quantify viable cells within a treated cellular suspension. In this procedure, normal and cancer cells were cultured in a 24-well plate at 130,000 cells per well. The supernatant was discarded after 24 h. The cells were gently separated and gathered using Trypsin–EDTA, as mentioned in the previous protocol^37^. After trypsin treatment, the collected cells were washed with PBS and resuspended in media. Subsequently, 0.4% 30 µl Trypan Blue dye was added to the cells and culture plates were incubated for 5 min at 37 °C. Using a hemacytometer, the number of living and dead cells was counted using a phase contrast inverted microscope. The cell viability was calculated using the following formula.

$$\% Cell\, Viability = \frac{Viable\, cell\, count\, \times 100}{{Total\, number\, of\, cells}}$$

#### Cytotoxicity analysis using MTT assay

MTT is a colorimetry assay which helps determine the administered treatment's cellular toxicity. To conduct the MTT assay as mentioned in our previous protocol, breast cancer cells (MCF-10A) and normal breast cells (MDA-MB-231) were seeded in a 96-well plate in the concentration of 1 × 10^6^ cells per well (100 µl). The media was replaced the next day, and 100 µl of crude drug and HA-coated 5-FU in ThCs-NPs dissolved in PBS at 10, 50 and 90 mg/ml concentrations were administered to cells^25^. The culture plates were incubated at 37 °C with 95% humidified air and 5% CO_2_ for 24 h. Control cells received PBS only; 10 µl MTT dye solution was added to cells and incubated for 4 h. Subsequently, the MTT dye was washed, and 100 µl DMSO was added to dissolve the formazan crystals, followed by incubation for 1 h again. A microplate reader was used at 560 nm to calculate the percentage of living and dead cells in the resulting colored solution using the following formula^42^.

$$Cell\, viability\, \left( \% \right) = \frac{OD\, of\, Cells\, treated\, with\, drug / NPs - OD\, blank\, \times 100}{{OD\, of\, control\, cells - OD\, of\, blank }}$$

#### Evaluation of 5-FU nanoparticle stability

The stability of the nanoparticle formulation was examined concerning its size, zeta potential, PDI, and morphology. After a storage period of three months in lyophilized form at 4 °C, the particles were subjected to analysis after reconstituting in deionized water at 37 °C.

## Statistical analysis

The outcomes of all investigations were subjected to statistical analysis utilizing a one-way analysis of variance (ANOVA) with a p-value of less than 0.05. The outcomes were then summarized, presenting the average three samples (*n* = 3), with standard deviation (SD).

## Results and interpretations

### Molecular docking of ligand HA with CD44 receptor

**Figure 2:**
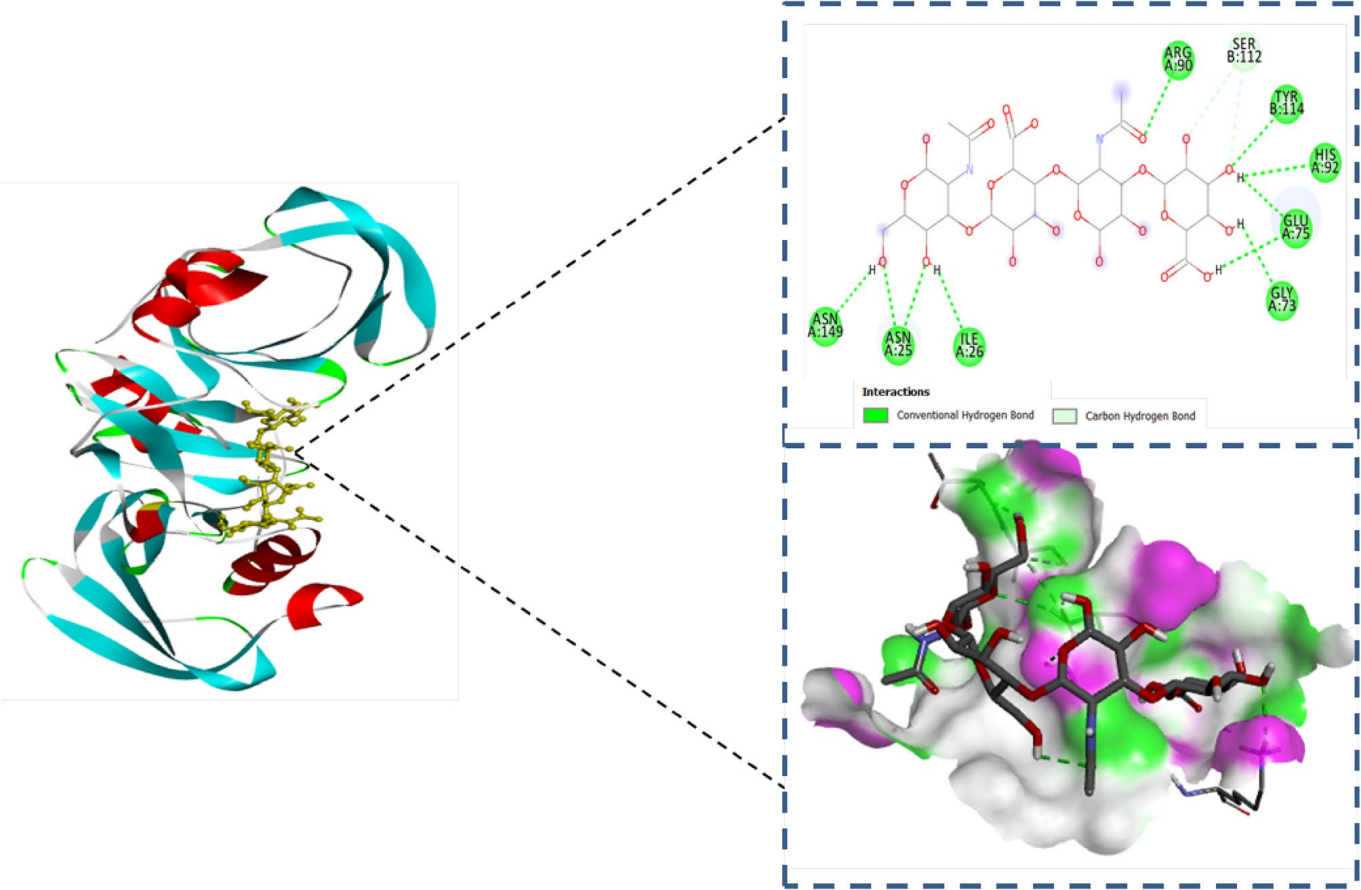
2d and 3d interactions of hyaluronic acid with CD44 macromolecule.

The molecular docking technique aids in predicting the binding affinity and interaction of ligands within the protein's binding site. Hyaluronic acid formed eight stable hydrogen bonds with target macromolecule CD44 at residues ASN A 25, ILE A 26, GLY A 73, GLU A 75, ARG A 90, HIS A 92, TYR B 114, and ASN A 149 and the binding energy was -8.5 kcal/mol. Conventional hydrogen bonds exhibited the hydrophilicity of the molecule, while the binding affinity determined the strength of interaction between macromolecule CD44 (PDB i.d 1UUH) and ligand HA (Fig. 2)^23^.

### Optimization of formulation

**Figure 3:**
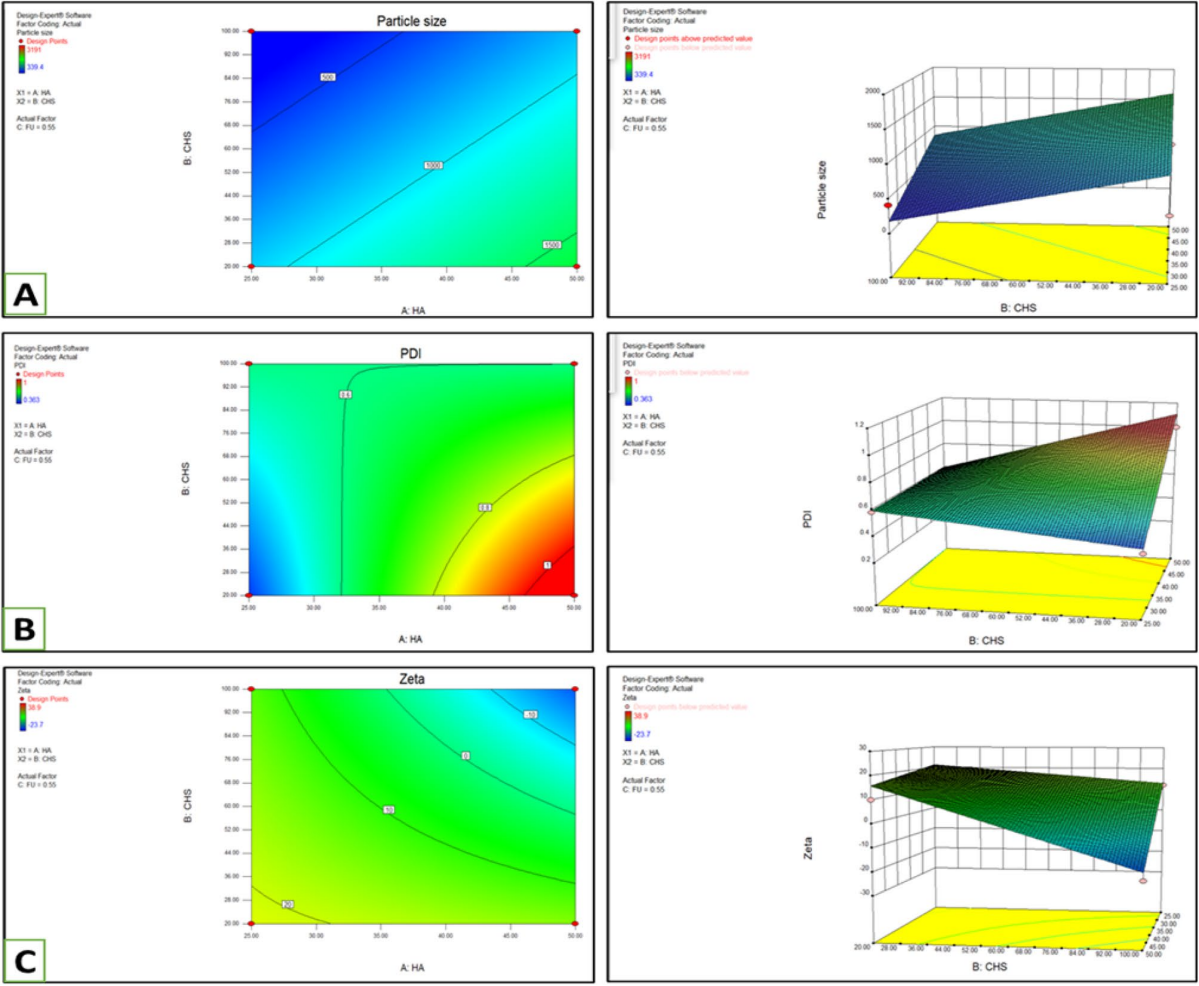
Nanoparticle size (**A**), PDI (**B**), and Zeta potential (**C**) considerations in a Box-Behnken factorial design using DOE using results by dependent parameters to optimize nanoparticle formulation parameters^24,35,38^.

Using Design of Expert, the BoxBehnken factorial design was employed to make the experimental trials affordable for the formulation of nanoparticles (Fig. 3). The particle size of nanoparticles, PDI, and ZP were used as dependent variables, and the concentration of HA, TCs and 5-FU were taken as dependent variables by keeping the concentration of TPP constant throughout all trials (Table 1). As illustrated in Table 2 and Fig. 3, optimal formulation parameters were selected to prepare the nanoparticles with minimum particle size, positive zeta potential, and uniform dispersity of particles within the matrix of the formulation.

**Table 2:** Particle size significantly affected by the concentration of TCs and HA.

Design summary											
File Version	8.0.6.1										
Study type	Response surface	Runs	12								
Design type	Box–Behnken	Blocks	No blocks								
Design mode	Quadratic	Build time	(r 0.75)								

Factor	Name	Units	Type	Subtype	Minimum	Maximum	Coded	Values	Mean	SD	
A	HA	mg	Numeric	Continuous	25.00	50.00	− 1.000 = 25.00	− 1.000 = 50.00	37.50	10.21	
B	CHS	mg	Numeric	Continuous	20.00	100.00	− 1.000 = 20.00	− 1.000 = 100.00	60.00	32.66	
C	FU	mg	Numeric	Continuous	0.10	1.00	− 1.000 = 0.10	− 1.000 = 1.00	0.55	0.37	

Response	Name	Units	Obs	Analysis	Minimum	Maximum	Mean	SD	Ratio	Trans	Model
yi	Particle size	nm	12	Polynomial	390.12	3191	899.018	838.877	8.17953	None	Linear
Y2	PDI		12	Polynomial	0.162	1	0.6645	0.276644	6.17284	None	2FI
Y3	Zeta	+/−mV	12	Polynomial	− 27.6	38.9	4.19667	20.6281	N/A	None	2FI

### Characterization of nanoparticles

#### Zeta analysis of HA-ThCs nanoparticles

**Figure 4:**
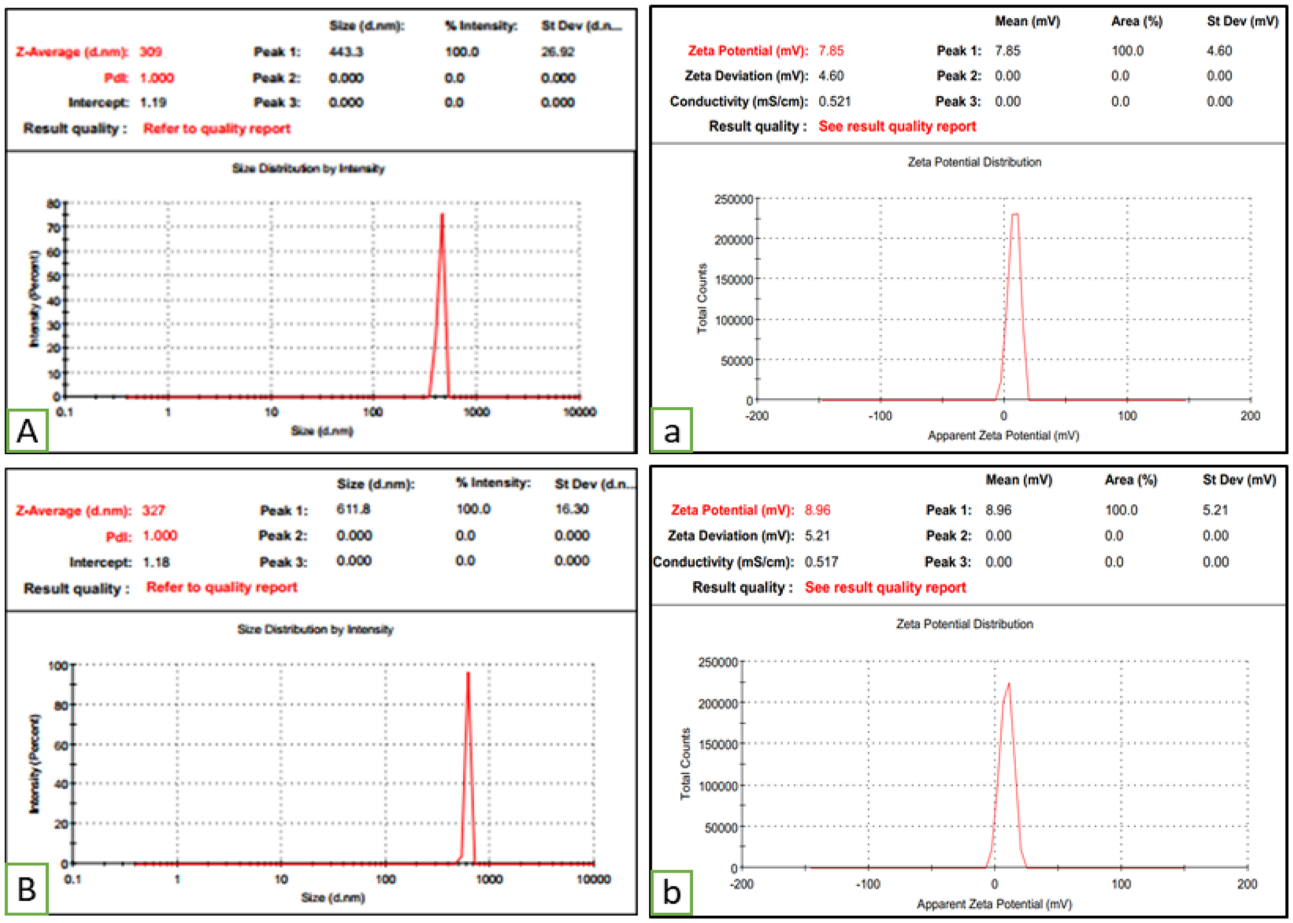
Particle size, matrix PDI (left) and zeta potential (right) of Empty HA-ThCs-NPs (**A**) and HA-coated 5-FU in ThCs-NPs (**B**) (mean + SD).

The results of Empty HA-ThCs-NPs and HA-coated 5-FU in ThCs-NPs in a triplicate manner are shown in Fig. 4 and Table 3. For Empty HA-ThCs-NPs, the mean smallest nanoparticle size was 309.2 nm, ZP 7.85 mV with PDI of 1.0. On the other hand, the HA-coated 5-FU in ThCs-NPs showed 327 nm, ZP 8.96 mV with PDI of 1.0. The results of PDI less than 1.0 show particles’ uniform dissemination in the nanoformulation. However, the results above 1.0 show heterogeneous diversity among particle sizes.

**Table 3:** Zeta analysis of prepared nanoparticles.

Sr	Formulations	Particle size (nm)	PDI	Zeta potential (mV)
1	Empty HA-ThCs-NPs	309	1.0	7.85
2	HA-coated 5-FU in ThCs-NPs	327	1.0	8.96

#### Morphological analysis

**Figure 5:**
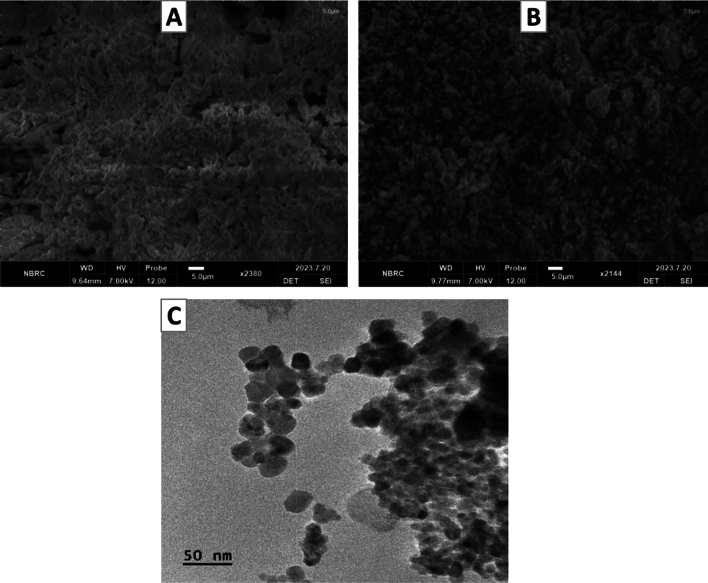
SEM images (**A**: upper) show spherical and smooth surfaces of nanoparticles of Empty HA-ThCs and (**B**: upper) HA-coated 5-FU in ThCs-NPs at the scale of 5.0um while the TEM images (**C**) showed more precise image of HA-coated 5-FU in ThCs-NPs at scale of 50 nm^24,35,38^.

The surface, uniformity, and size of the nanoparticles of Empty and 5-FU loaded formulations were analyzed using SEM and TEM examination. The observations showed that the Empty HA-ThCs-NPs and HA-coated 5-FU in ThCs-NPs showed smooth surfaces with a uniform distribution of particles, as illustrated in Fig. 5. Regardless of the different routes of administration for therapeutic moieties, the particle size and shape have a protruding influence on the transport rate and diffusion mechanism of nanoparticles inside the cell matrix.

#### Functional group identification

**Figure 6:**
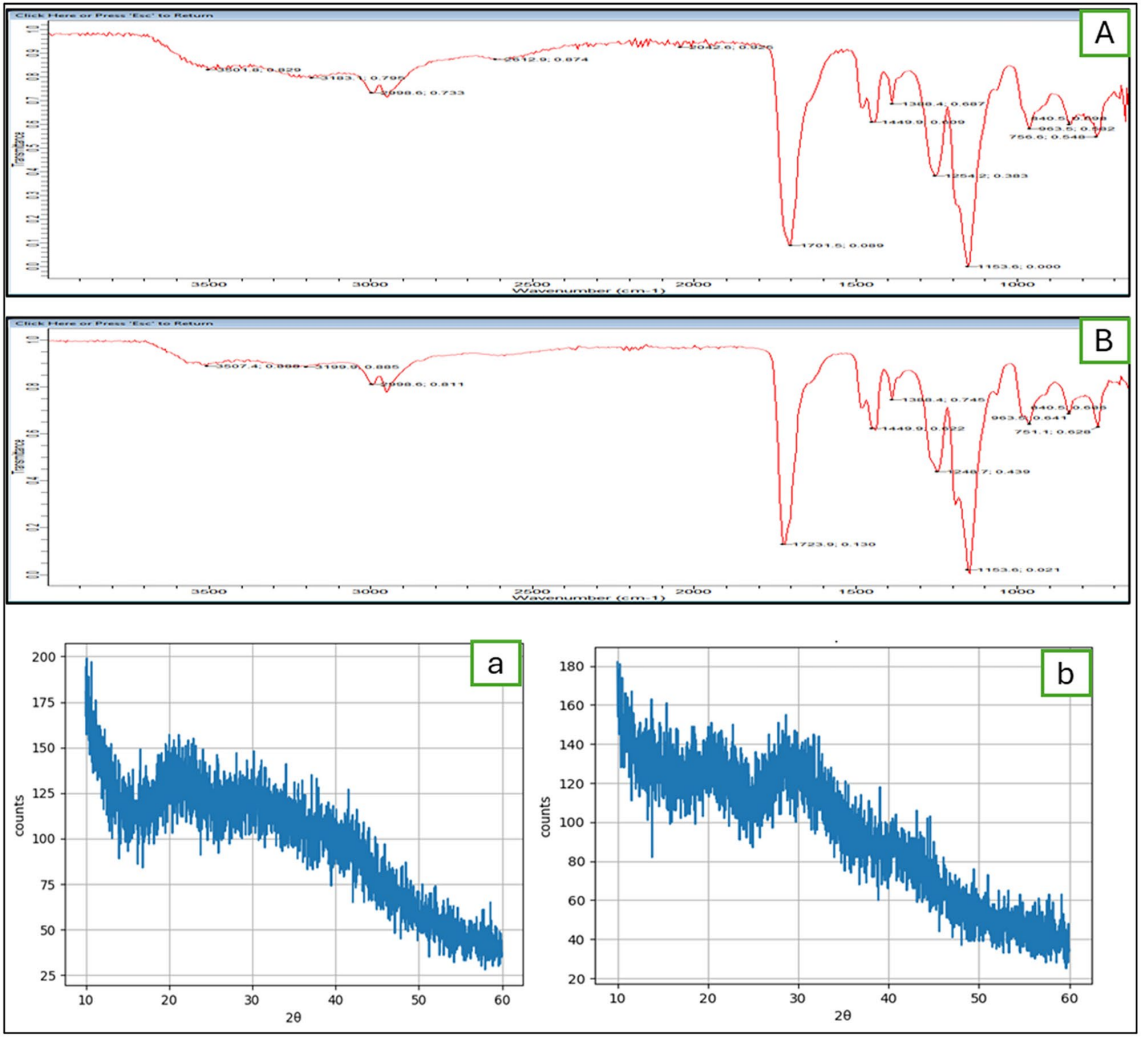
Functional group analysis of (**A**, a) Empty HA-ThCs and (**B**, b) HA-coated 5-FU in ThCs-NPs (Upper graphs for FTIR and lower graphs showed XRD analysis)^24,35,38^.

The FTIR spectra directed the manifestation of representative points and evidence about the phase composition of 5-FU-loaded nanoparticles compared with the empty formulation in the lyophilized form, as illustrated in Fig. 6. The prominent bands were detected at 3480 cm^−1^ in line with the widening of hydroxyl (–OH) groups, at 2925–2930 cm^−1^ in line with the widening of methyl (-CH), at 1625 to 1655 cm^−1^ due to the stretching of amide (−C = O), at 1275 to 1280 cm^−1^ due to the presence of carbonyl (−CN) bonds.

The properties of intermolecular and extra-molecular interactions on the amorphous surface of Empty and HA-coated 5-FU in ThCs-NPs were examined with X-ray diffraction analysis (XRD). The protruding reflection was positioned at 2θ = 14.8◦, and significantly fewer were shown at 22.25° in Empty and HA-coated 5-FU in ThCs-NPs, showing almost similar functional groups indicated by FTIR in Fig. 6.

#### Raman spectroscopy

**Figure 7:**
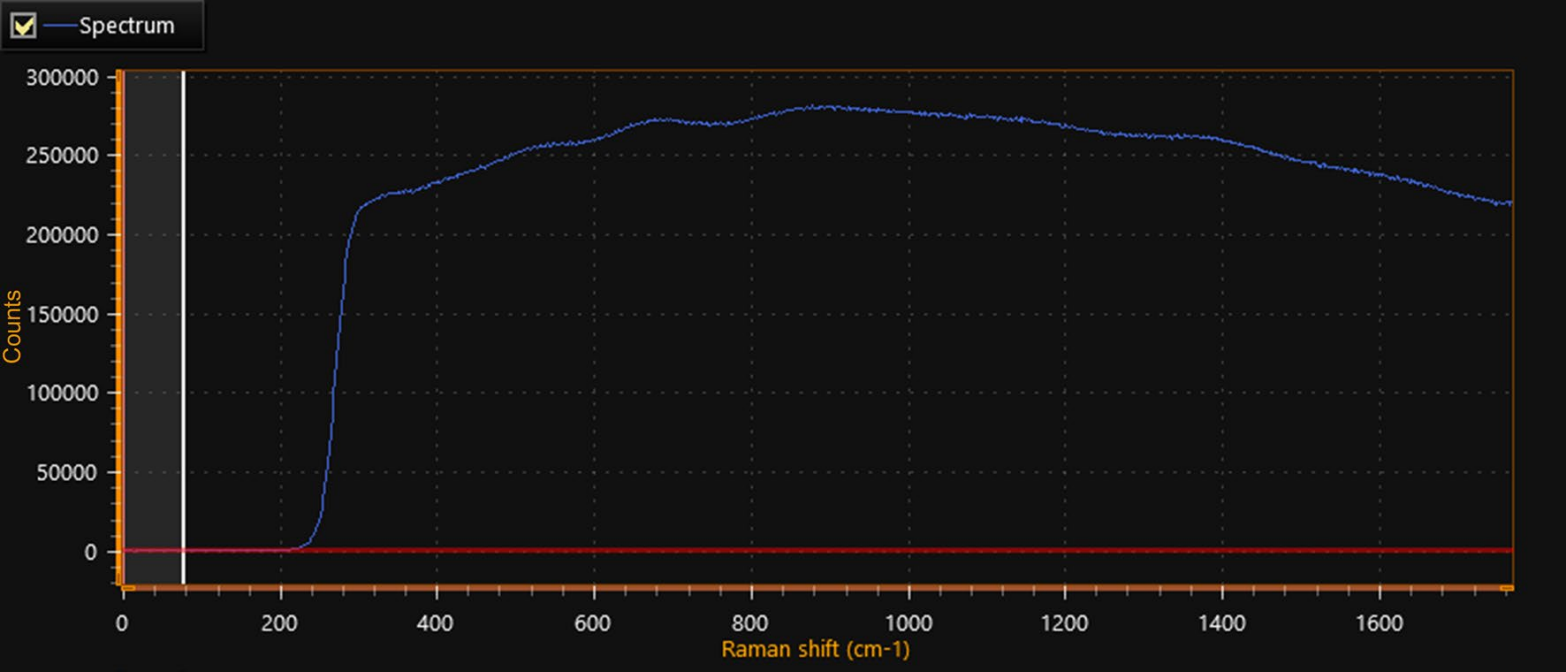
Raman analysis of at 50X HA-coated 5-FU in ThCs-NPs.

Raman spectroscopy results for HA-coated 5-FU in ThCs-NPs (Fig. 7) revealed that nanoparticles have porous surfaces, so they showed deflection in the magnetic field, making them ideal for a drug delivery system. The drug is successfully encapsulated in the nanoparticles with uniform distribution.

#### Percentage of drug loading (DL) and encapsulation efficiency (EE)

UV–Vis spectrophotometer was used to calculate the quantity of loaded 5-FU in the nanoparticles. According to Table 4, the average percentages of EE and DL in the nanoparticles of 5-FU encapsulated in ThC-HA were 82% and 19%, respectively.

**Table 4:** Percentages of EE and DL of HA-coated 5-FU in ThCs-NPs.

Absorbance	Encapsulation efficiency (EE%)	Encapsulation efficiency % (mean ± SD)	Drug loading (DL)	Drug loading % (mean ± SD)
2.903	81.35	82.3% ± 0.01	19.32	19.27 ± 0.05
2.818	82.78		20.22	
2.781	81.97		18.59	

#### 5-FU release in in vitro assembly

**Figure 8:**
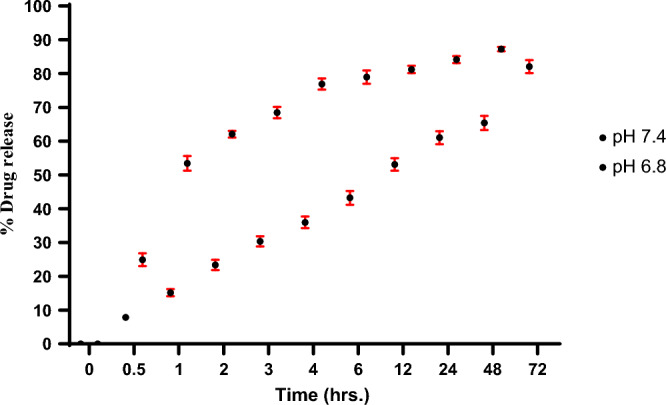
The drug release percentages from nanoparticles containing 5-FU were evaluated in PBS at pH 6.8 and 7.4 over specific time intervals. Statistical analyses were conducted using one-way ANOVA and a t-test, with a significance level set at *p* < 0.05. The results are the mean ± standard deviation (*n* = 3).

The drug encapsulated in nanoparticles was released from the dialysis bag assembly immersed in PBS at pH 7.4 and 6.8 at room temperature. Table 1 (supplementary material) and Fig. 8 represent the time-dependent average values of percentage drug release, and they show that 5-FU from nanoparticles was released up to 72 h at pH 7.4 (mimicking the physiological environment of the human body). The drug release from nanoparticles was around 85% in an acidic medium, a comparatively improved and sustained release.

#### Releasing pattern of 5-FU from nanoparticles

**Figure 9:**
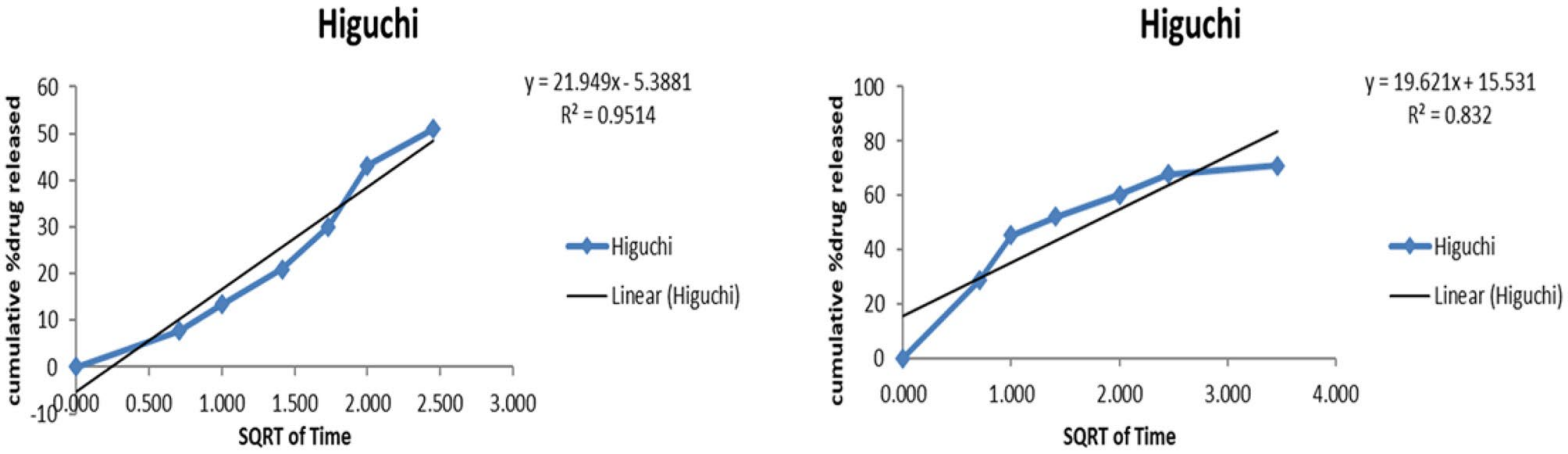
Kinetic models on 5-FU released from nanoparticles at pH 7.4 (**A**) and 6.8 (**B**) following Higuchi diffusion model^23,24,35^.

The kinetic parameters using the information of the cumulative percentage of 5-FU release at pH 7.4 and 6.8 over specific time intervals were used to study the release pattern, as stated in Table 2. The Higuchi diffusion model is better than all kinetic models at both pHs. The reason for selecting these particular kinetic parameters is that their R^2^ values were close to one, indicating a solid fit to the Higuchi diffusion model for drug release. Table 3 (supplementary material) and Fig. 9 display the drug release rate (R^2^) and the corresponding slope values (k) for all the kinetic parameters analyzed. The relatively slow drug release can be attributed to the stability of the network model of the nanoparticles at both pH levels.

#### In vitro anticancer potential of nanoparticles

### MTT assay for cell viability

**Figure 10:**
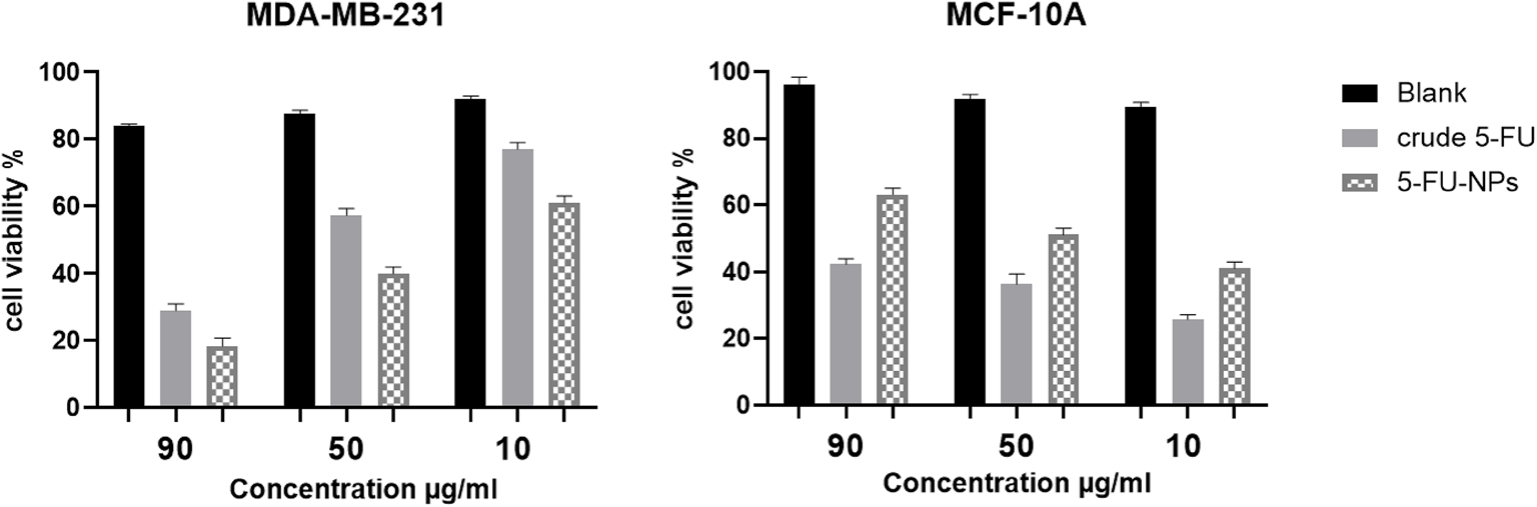
The observations of cell viability analysis using the MTT assay for MDA-MB-231 and MCF-10A cells after exposure to 5-FU-NPs were compared to those treated with crude 5-FU. The data is presented as the mean ± standard deviation (*n* = 3), and statistical significance was achieved with a significance level set at *p* < 0.05.

The anticancer potential of 5-FU-NPs was tested on MDA-MB-231 and MCF-10A at concentrations of 10, 30, and 90 µg/ml compared to crude 5-FU (Fig. 10). When the drug concentration was increased from 10 to 50 and 90 µg/ml, it had a dose-dependent effect on the cell survival of MCF-10A cells exposed to crude form and 5-FU-NPs.

When MDA-MB-231 cells were exposed to crude 5-FU, the highest observed cell viability was 77.31 ± 0.02% at a concentration of 10 µg/ml, while the lowest viability was 29.8 ± 0.05% at 90 µg/ml, resulting in an IC50 value of 35.7 ± 0.02%. In contrast, treatment with 5-FU-NPs significantly decreased the viability of these cancer cells. At a concentration of 10 µg/ml, the viability was 61.88 ± 0.11%, and it dropped to 17.97 ± 0.10% at 90 µg/ml, with an IC50 of 42.1 ± 0.11 µg/ml for MDA-MB-231 cells.

For MCF-10A cells, exposure to crude 5-FU resulted in the highest cell viability of 29.78 ± 0.14% at 10 µg/ml and the lowest viability of 14.39 ± 0.03% at 90 µg/ml, with an IC50 value of 25.3 ± 0.04%. When treated with 5-FU-NPs, cell viability was less significantly reduced. At 10 µg/ml, the viability was 41.21 ± 0.11%, and at 90 µg/ml, it was 63.21 ± 0.10%, with an IC50 of 51.3 ± 0.04 µg/ml for MCF-10A cells.

The synthesized 5-FU-NPs were less toxic to normal breast cancer cells (MCF-10A) than crude 5-FU. However, they exhibited high cytotoxicity towards cancerous cells (MDA-MB-231) compared to crude 5-FU. This suggests that the 5-FU-NPs may offer a more targeted and effective treatment approach for cancer cells while sparing normal cells.

### Trypan blue exclusion assay for cytotoxicity

We used the Trypan blue exclusion assay to assess the cytotoxic potential of 5-FU-NPs compared to crude 5-FU at different concentrations of 10, 50, and 90 µg/ml. The data presented in Table 5 demonstrates that the percentage of cytotoxicity increased with higher concentrations of nanoparticles. For MDA-MB-231 cells, the highest cytotoxicity, at 77.8 ± 0.04%, was observed at a concentration of 90 µg/ml, while the lowest cell viability, at 10 ± 0.01%, was found at a concentration of 0 µg/ml when treated with crude 5-FU.

**Table 5:** Trypan blue exclusion result of % cytotoxicity at 10, 50, and 90 μg/ml concentrations (*p*< 0.05 mean ± SD).

S.No	Concentration	HA-coated 5-FU in ThCs-NPs	Crude-5-FU
%Cytotoxicity MDA-MB-231			
1	90 µg/ml	85±0.04	77.8±0.04
2	50 µg/ml	60.3±0.21	53.6±0.04
3	10 µg/ml	35.3±0.03	32.1±0.12
4	0 µg/ml	10±0.07	10±0.01
%Cytotoxicity MCF-10A			
1	90 µg/ml	25±0.03	67±0.02
2	50 µg/ml	18.4±0.02	46±0.03
3	10 µg/ml	12±0.05	29.7±0.04
4	0 µg/ml	10±0.07	11±0.04

Similarly, for MCF-10A cells, the highest cytotoxicity, at 85 ± 0.04%, was observed at a concentration of 90 µg/ml when treated with 5-FU-NPs, while the lowest cell viability, at 10 ± 0.07%, was noted at the same concentration (90 µg/ml) for 5-FU-NPs treated cells.

### Cell morphology analysis for cytopathic effect

**Figure 11:**
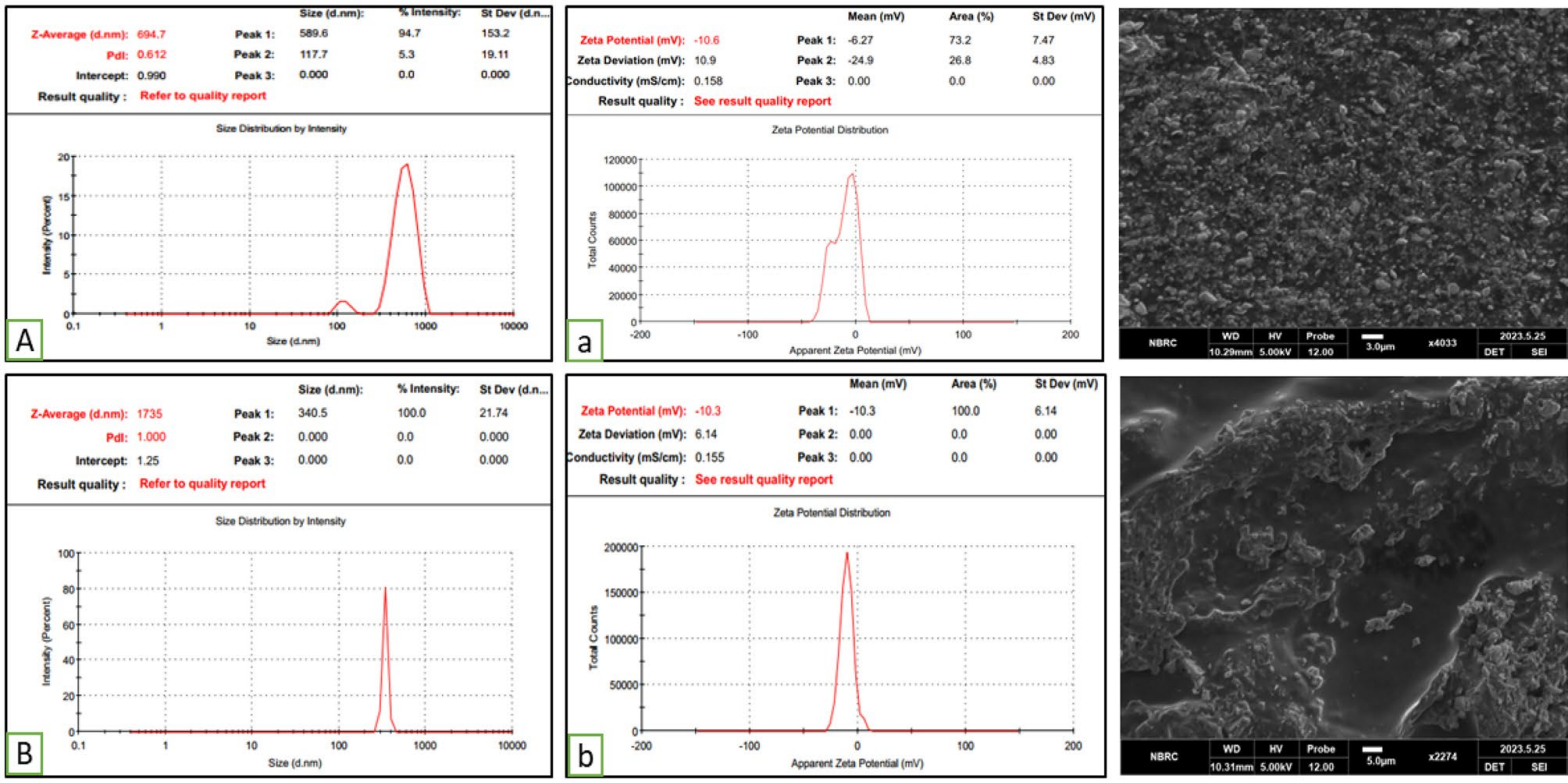
Reconstituted extemporaneous liquid formulation of HA-coated 5-FU in ThCs-NPs (**A**) particle size, PDI and zeta potential (left) and SEM analysis (right). Liquid formulation stored for 3 months (**B**) particle size, PDI and zeta potential (left) and SEM analysis (right) (mean + SD)^24,35,38^.

The changes in cellular changes, characterized by clumping, aggregation, blobbing, rounding and detachment from the flask, are the characteristic features of apoptosis, representing that the treatment rendered a cytotoxic effect on cells. As observed for MCF-10A cells, the 5-FU NPs did not induce any cytotoxic effect on cells, even at high doses of 90 µg/ml at 12 and 24-h intervals. In contrast, pure 5-FU showed no cytopathic effect on 10 µg/ml at both intervals (12 and 24 h). An insignificant change in characteristic cellular morphology and detachment can be seen at 50 µg/ml, which is more evident at a time interval of 24 h, whereas at 90 µg/ml, rounding and aggregation of cells are more profound, which shows that pure 5-FU induces cytotoxicity in MCF-10A cells (Fig. 11).

### Stability studies

The stability of HA-coated 5-FU within ThCs-NPs was assessed by monitoring changes in particle size, shape, PDI, and zeta potential over three months under storage conditions of 4 °C and ambient temperature.The characterization of the reconstituted extemporaneous liquid formulation revealed a slight alteration in particle size, which increased from 69.7 nm, with the PDI shifting from 0.6, and the zeta potential changing from − 10.6 mV, as depicted in Fig. 11A (left). Additionally, SEM analysis indicated that the nanoparticles maintained their smooth spherical shape (right). The encapsulation efficiency and drug loading of this formulation experienced a slight decrease, reducing to 77.47 ± 0.29% from the initial 82.3 ± 0.01% and to 17.3 ± 0.27% from 19.27 ± 0.05%.

However, when the characterization was performed for nanoparticles stored in a liquid form, significant changes were observed in particle size, which increased with 1735 nm, with the PDI moving from 1 and the zeta potential shifting − 10.3 mV, as illustrated in Fig. 11B (left). In this case, the nanoparticles displayed a rough, non-spherical shape (right). Consequently, storing the nanoparticles in a dried powder is recommended for long-term use to maintain their stability.

## Discussion

Breast cancer remains a significant global public health challenge and is currently the most common cancer type worldwide. The increased awareness of breast cancer heightened public attention, and advancements in breast imaging have positively impacted early detection and screening for this disease. Breast cancer is a life-threatening condition for women and is the primary cause of death among females^43^. The present study describes the synthesis and characterization of an advanced drug delivery platform for tumor therapy to make the treatment effective, especially for breast cancer. The results of this study state that the nanoformulation with the modified polymers augment targeted drug delivery compared to the conventional chemotherapeutic drug^44^. In the current research, 5-fluorouracil (5-FU) was used against breast cancer cell line. In breast cancer treatment, 5-fluorouracil is often combined with other chemotherapy drugs to maximize its effectiveness. the study showed that the emergence of drug resistance and dose-limiting cytotoxicity are substantial obstacles to successfully utilizing 5-FU^12^.

In a recent study, researchers developed a nanocomposite by crosslinking chitosan and agarose to form a polymeric hydrogel. They incorporated γ-alumina nanoparticles within the hydrogel to deliver 5-FU. The nanocomposite was encapsulated in a water-in-oil-in-water emulsion system. Tests using breast cancer cells (MCF-7) showed that the 5-FU-loaded nanoemulsion eliminated cancerous cells more effectively than crude 5-FU^45^. Similarly, in a 2021 study published in Nanoscale Research Letters, hyaluronic acid (HA)-functionalized regenerated silk fibroin-based nanoparticles (NPs) were used to simultaneously deliver curcumin (CUR) and 5-FU to breast tumor cells. Various weight ratios of CUR to 5-FU were tested, and their combined delivery demonstrated potential for breast cancer treatment^46^.

To achieve the primary goal of this study, which involves modification of the polymer, i.e. (thiolation of chitosan) and subsequently developing formulation using the thiomer, we employed the EDAC coupling mechanism. The process involved the preparation of TCS by creating an amide bond between the amino group of the polymer and the carboxylic group of TGA^18^. To use HA for surface functionalization, we observed the molecular docking of Ligand HA and with CD44 receptor macromolecule. in-slico analysis indicated eight stable hydrogen bonds and the bonding showed the hydrophilicity of the molecules. At the same time, the strength of interaction between the molecules is determined by binding affinity^20^. Our results follow previous reports exploring HA-derived conjugates and nanoparticles to target the CD44 receptor on cells to precisely deliver therapeutics and imaging agents^7^.

The formulation of hyaluronic acid-coated thiolated chitosan nanoparticles, followed by loading 5-FU to the above formulation, was successfully synthesized by ion gelation^46^. The purpose of selecting this method is its simplicity and ease of preparation. The same techniques were adopted by Shahnaz and coworkers to form nanocarriers for the oral and nasal administration of leuprolide^47^.

The next step in the present study was optimizing the formulation, which was achieved by using the Box Behkxen factorial design to make the experimental trials affordable for the formulation of nanoparticles (Fig. 2). The optimized formulation was selected for further evaluation^48^. The optimized formulation had a size of 339.4 nm and 0.36 PDI, with no aggregated or large particulate structures and 18.1 zeta potential. The drug 5-FU has been reduced to nanoparticles to better target the cancer cells. Zeta sizing predicts the size and stability of prepared nanoformulations^49^. The low PDI (less than 0.5), as illustrated in Table 3, showed uniform size distribution and stability in nanocarrier formulation. The zeta potential is a measure of the stability of nanoformulations, and a positive charge on zeta potential depicted the cationic nature of the nanoparticulate system in this study. The physical stability of particle dispersion can be assessed or predicted by Zeta potential; moreover, a higher ZP delivers the electrostatic repulsion force^50,51^.

The present study confirmed a successful nanoformulation, as no interaction between the drug and the modified polymer was shown through the FTIR spectrum. In this analysis, characteristic peaks in the final formulation provide compelling evidence of successful drug incorporation into the polymeric nanoparticles. Furthermore, no apparent interaction between the drug-polymer complex was observed, as indicated by the unique peaks corresponding to functional groups in the FTIR spectrum (Fig. 6a).

An amide bond formation was evident from the interaction between the CS polymer and TGA, as evidenced by the complementary peaks related to thiol groups in the spectrum of thiolated chitosan. Additionally, S–S disulfide bond formation was confirmed from a peak at 1000 cm^−1^. The establishment of C–NH amide bond formation was supported by the presence of C = O stretching at 1645 cm^−1^ and the alteration in the N–H extending signal at 3225 cm^−1^, compared to pure CS. Furthermore, the existence of thiol groups was verified by observing the -SH stretching peak at 2496 cm^−1^^47^. Additional analysis was conducted using X-ray diffraction, as shown in Fig. 6b. Results of XRD images integrating modified polymer revealed an absence of the characteristic peaks observed in the drug. This observation suggests that the formulation has transformed the drug from its original crystalline state into an amorphous one. This finding offers compelling evidence of the polymer's transition from a crystalline structure to an amorphous one during the formulation process^44,48^.

In this study, we investigated the morphology of our formulations using SEM and TEM imaging techniques. As depicted in Fig. 5a, the SEM image of 5-FU loaded HA-coated ThCs-NPs exhibited concentrated spherical nanoparticles. Similarly, the smaller size of the polymeric formulation, as indicated by zeta sizing results, was further confirmed through TEM analysis, which shows spherical nanoparticles as depicted in Fig. 5b. In the 5-FU loaded HA-coated ThCs-NPs, SEM imaging revealed uniform spherical-shaped nanoparticles. TEM observations also confirmed that the nanoparticles, formulated with 5-FU loaded HA-coated ThCs-NPs, exhibited a smooth surface and a spherical shape. This spherical morphology contributes to the stability of the polymeric system^52^.

In the present study, the amount of 5-FU loaded in the nanoparticles was determined by a UV–Vis spectrophotometer. The average percentages of EE and DL in the nanoparticles of 5-FU encapsulated in ThC-HA were 82% and 19%, respectively, as depicted in Table 4. The encapsulation capacity allows for the controlled and sustained release of drugs over an extended duration within the body. Additionally, polymeric NPs facilitate the attachment of multiple ligands, enabling the creation of versatile colloidal formulations. Consequently, these advanced particulate systems present numerous opportunities for advancing precise tumor-targeting strategies^53^. A study showed that 5-FU-loaded nanoparticle formulated by including PVA as an internal aqueous phase stabilizer has significantly influenced NP size, encapsulation efficiency, and the initial burst release^23^. Another study has shown that the hydrophilic nature of 5-FU results in its leakage into the external aqueous phase during the initial stages of particle formation, ultimately leading to reduced encapsulation efficiencies^14,19^.

In vitro, the drug release profile of nanoparticles in phosphate buffers at pH 7.4 and 6.8, maintained at 37 °C (Table 1), displayed time-dependent mean percentage release, revealing that 5-FU was released from the nanoparticles over 72 h at pH 7.4. Conversely, in a slightly acidic environment, the drug release from the nanoparticles reached approximately 84.7%, signifying a more effective and sustained release^24^. Table 2 showcases various mathematical models for predicting drug release kinetics, encompassing zero order, first order, Higuchi, Korsmeyer-Peppas, and Hixon Crowell models. The release of 5-FU nanoparticles exhibited a superior fit with the Higuchi model (R^2^ = 0.9864). This model characterizes drug release through non-erodible diffusion-based mechanisms within a matrix^54^. Therefore, it can be inferred that the release of the drug solution occurred at a relatively gradual rate due to the stable network model of the nanoparticles at both pH levels. The stable network model of the nanoparticles comparatively slows down the drug release rate at a specific pH. A prior study explored the release of polypeptide drugs from CHS–CS nanoparticles targeting the colon and revealed the pH dependence of the process^26^.

In contrast to the crude 5-FU, the synthesized 5-FU nanoparticles displayed lower toxicity towards normal breast cancer cells but exhibited higher cytotoxicity against cancerous cells. Figure 10 depicted that the 5-FU nanoparticles had the most significant cytopathic effect (CPE) on MDA-MB-231 cells, comparable to the results observed with crude 5-FU. Contrariwise, when treating MCF-10A cells with a concentration of 90 µg/ml, crude 5-FU exhibited the highest CPE, while 5-FU nanoparticles had a limited cytopathic effect on MCF-10A cells. These observations regarding cytotoxicity and cytopathic effects highlight the nanoparticles' potent and safer anticancer activity than the crude drug^55^.

## Conclusion

The present study selected Hyaluronic acid as the ligand to transport the chemotherapeutic agent, 5-Fluorouracil, encapsulated within the natural polymer compound, thiolated chitosan. This choice was made based on several advantageous properties, such as hydrophilicity, low immunogenicity, biocompatibility, and numerous modifiable functional groups. This particular formulation was achieved through the ionic gelation method, with the primary goal of improving targeted drug delivery to enhance the efficiency of the chemotherapy by modifying the nanoparticle surface with a ligand. These formulated nanoparticles exhibited a particle size ranging from 200 to 300 nm and a positively charged zeta potential, facilitating their uptake by negatively charged cancer cells. They also displayed good stability and achieved high encapsulation efficiency. Our in vitro efficacy evaluation assessed the nanoparticles on normal epithelial breast cells (MCF-10A) and epithelial breast cancer cells (MDA-MB-231). These nanoparticles demonstrated significant cytotoxicity compared to raw 5-FU (*p* < 0.05) and showed an excellent release profile following a simple diffusion model. These characteristics collectively enhance these nanoparticles' pharmacokinetic profile, effectiveness, and active targeting capabilities, positioning them as a promising targeted therapeutic option for breast cancer treatment. Nevertheless, further in vivo assessments of these formulations are crucial to validate these findings.

### Supplementary Information

Supplementary Tables.

## Data Availability

Data is available in the manuscript file.
