# Publisher Correction: Enhancing therapeutic efficacy: sustained delivery of 5-fluorouracil (5-FU) via thiolated chitosan nanoparticles targeting CD44 in triple-negative breast cancer

**DOI:** 10.1038/s41598-024-67606-5

**Published:** 2024-07-25

**Authors:** Sadia Anjum, Faiza Naseer, Tahir Ahmad, Faryal Jahan, Halima Qadir, Rabia Gul, Kousain Kousar, Atif Sarwar, Abdallah Shabbir

**Affiliations:** 1 Department of Biology, University of Hail, Hail, Saudi Arabia; 2 Department of Biosciences, Shifa Tameer e Millat University, Islamabad, Pakistan; 3 Industrial Biotechnology, Atta-ur-Rahman School of Applied Biosciences, National University of Sciences and Technology, Islamabad, Pakistan; 4 Shifa College of Pharmaceutical Sciences, Shifa Tameer e Millat University, Islamabad, Pakistan

Correction to: *Scientific Reports* 10.1038/s41598-024-55900-1, published online 19 May 2024

The original version of this Article contained errors.

In the Materials and Methods Section, Under the Subheading ‘In Silico Analysis’, the Subsection ‘Calibration Plot for 5-FU in Distilled Water and Phosphate Buffer at pH 7.4’ has been added.

Under the subheading ‘In vitro analysis: release of 5-FU from nanoparticles’,

“The experiment was performed in triplicate, ensuring robust and reliable outcomes^37,38^.”

now reads:

“The experiment was performed in triplicate, ensuring robust and reliable outcomes^23,37^.”

In the Results and interpretations section, under the subheading ‘Zeta analysis of HA-ThCs nanoparticles’,

“The results of Empty HA-ThCs-NPs and HA-coated 5-FU in ThCs-NPs in a triplicate manner are shown in Fig. 4 and Table 3. For Empty HA-ThCs-NPs, the mean smallest nanoparticle size was 396.2 nm, ZP 15.1 mV with PDI of 0.52. On the other hand, the HA-coated 5-FU in ThCs-NPs showed 339.4 nm, ZP 18.1 mV with PDI of 0.36. The results of PDI less than 0.5 show particles' uniform dissemination in the nanoformulation. However, the results above 0.5 show heterogeneous diversity among particle sizes.”

now reads:

“The results of Empty HA-ThCs-NPs and HA-coated 5-FU in ThCs-NPs in a triplicate manner are shown in Figure 4 and Table 3. For Empty HA-ThCs-NPs, the mean smallest nanoparticle size was 309.2 nm, ZP 7.85 mV with PDI of 1.0. On the other hand, the HA-coated 5-FU in ThCs-NPs showed 327 nm, ZP 8.96 mV with PDI of 1.0. The results of PDI less than 1.0 show particles' uniform dissemination in the nanoformulation. However, the results above 1.0 show heterogeneous diversity among particle sizes.”

Under the subheading ‘Stability studies’,

“The characterization of the reconstituted extemporaneous liquid formulation revealed a slight alteration in particle size, which increased from 339.9 nm to 387.3 nm, with the PDI shifting from 0.36 to 0.59, and the zeta potential changing from 18.1 to 16.4 mV, as depicted in Fig. 11A (left).”

now reads:

“The characterization of the reconstituted extemporaneous liquid formulation revealed a slight alteration in particle size, which increased from 69.7 nm, with the PDI shifting from 0.6, and the zeta potential changing from − 10.6 mV, as depicted in Figure 11A (left).”

“However, when the characterization was performed for nanoparticles stored in a liquid form, significant changes were observed in particle size, which increased from 339.9 nm to 614.3 nm, with the PDI moving from 0.36 to 0.40 and the zeta potential shifting from 18.1 to − 23.7 mV, as illustrated in Fig. 11B (left).”

now reads:

“However, when the characterization was performed for nanoparticles stored in a liquid form, significant changes were observed in particle size, which increased with 1735 nm, with the PDI moving from 1 and the zeta potential shifting − 10.3 mV, as illustrated in Figure 11B (left).”

In addition, Tables 1 and 3 contained errors. In Table 1, the “Particle size nm”, “PDI” and “Zeta Potential +/−mV” for Run 5 were incorrect. The incorrect and correct values appear below.

Incorrect:

**Table:** 

Run	HA (mg)	TC (mg)	5-FU (mg)	Particle size (nm)	PDI	Zeta potential ( +/−mV)
**5**	**25.00**	**60.00**	**0.10**	**339.4**	**0.36**	**18.1**

Correct:

**Table:** 

Run	HA (mg)	TC (mg)	5-FU (mg)	Particle size (nm)	PDI	Zeta potential ( +/−mV)
**5**	**25.00**	**60.00**	**0.10**	**327**	**1**	**8.96**

In Table 3, the “Particle Size (nm)”, “PDI” and “Zeta Potential (mV)” were incorrect. The incorrect and correct values appear below.

Incorrect:

**Table:** 

Sr	Formulations	Particle size (nm)	PDI	Zeta potential (mV)
1	Empty HA-ThCs-NPs	396.2	0.52	15.1
2	HA-coated 5-FU in ThCs-NPs	339.4	0.36	18.1

Correct:

**Table:** 

Sr	Formulations	Particle size (nm)	PDI	Zeta potential (mV)
1	Empty HA-ThCs-NPs	309	1.0	7.85
2	HA-coated 5-FU in ThCs-NPs	327	1.0	8.96

Additionally, Figures 4, 5, 9 and 11 contained errors due to the aforementioned wrong values. The original Figures and accompanying legends appear below.

**Figure 4:**
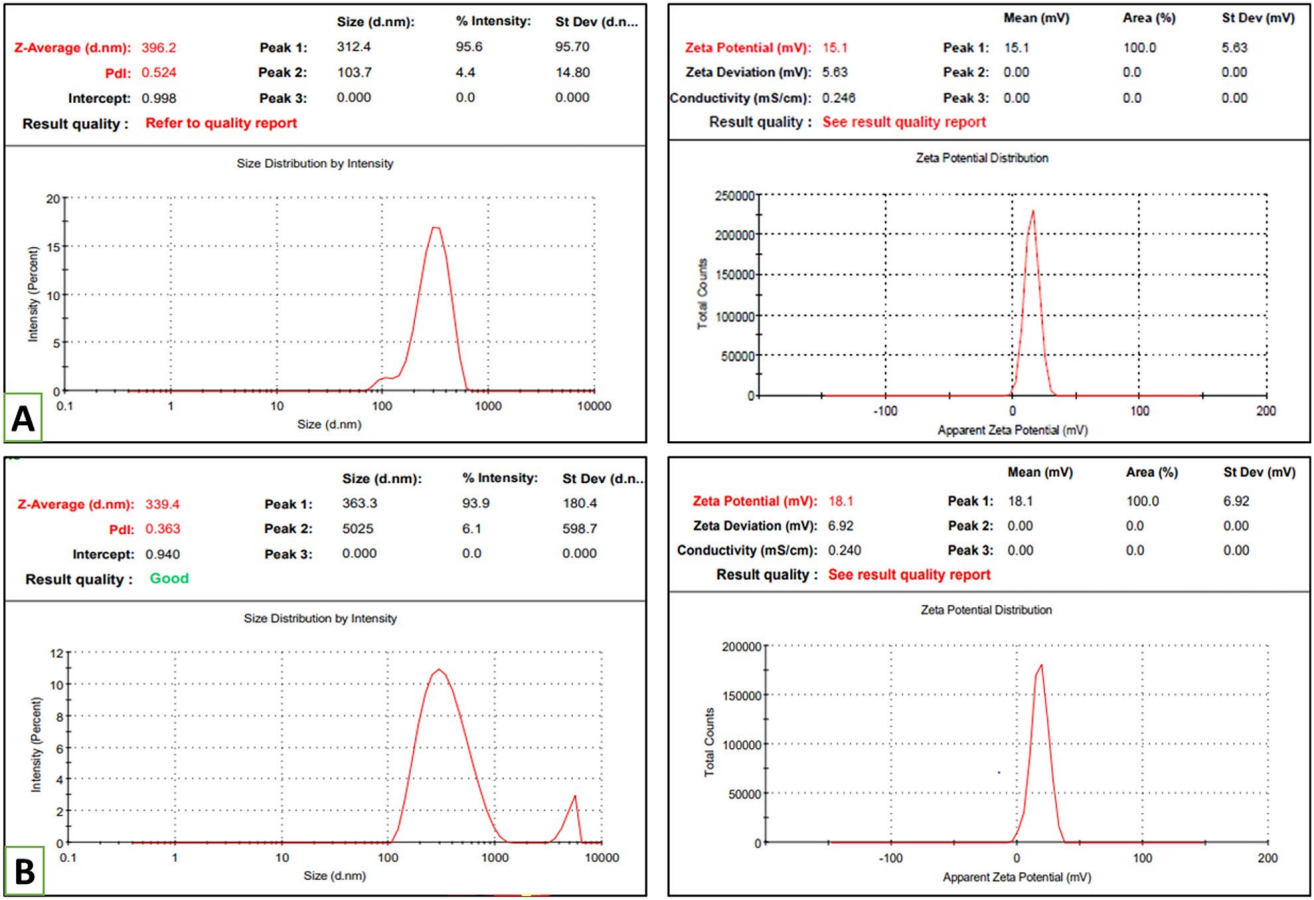
Particle size, matrix PDI (left) and zeta potential (right) of Empty HA-ThCs-NPs (**A**) and HA-coated 5-FU in ThCs-NPs (**B**) (mean + SD).

**Figure 5:**
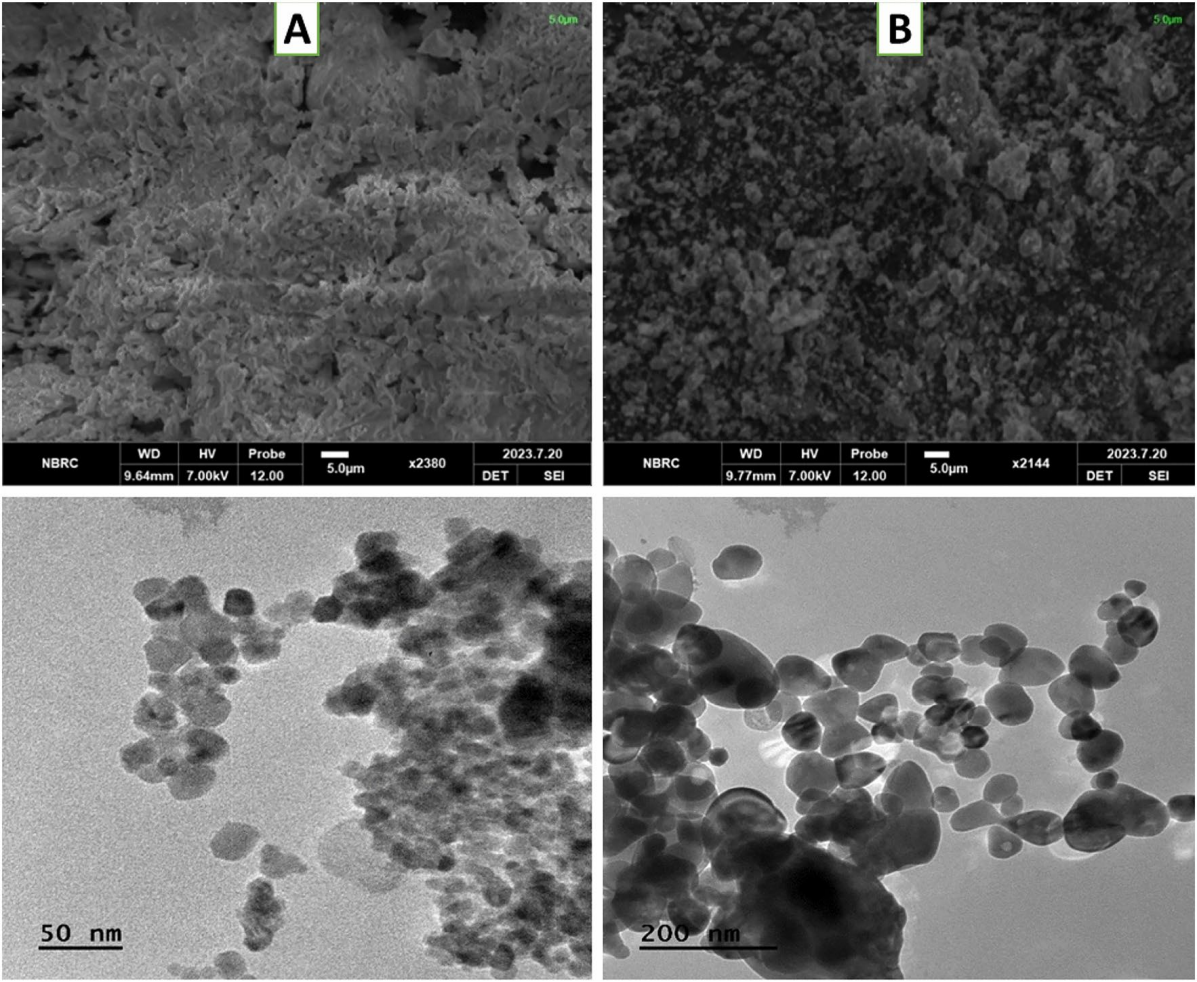
SEM images (**A**: upper) show spherical and smooth surfaces of nanoparticles of Empty HA-ThCs and (**B**: upper) HA-coated 5-FU in ThCs-NPs at the scale of 5.0um while the TEM images (**A**: lower) showed more precise images of spherical and smooth surfaces of Empty HA-ThCs and (**B**: lower) HA-coated 5-FU in ThCs-NPs at scale of 50 nm and 200 nm^23,24,35^.

**Figure 9:**
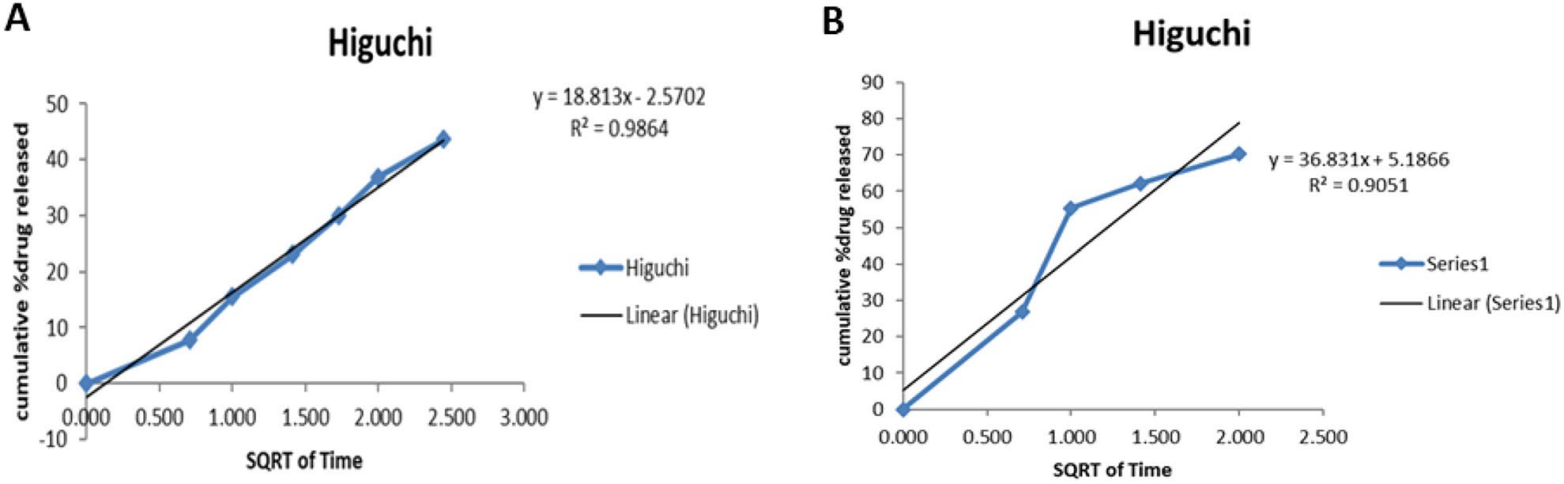
Kinetic models on 5-FU released from nanoparticles at pH 7.4 (**A**) and 6.8 (**B**) following Higuchi diffusion model^23,24,35^.

**Figure 11:**
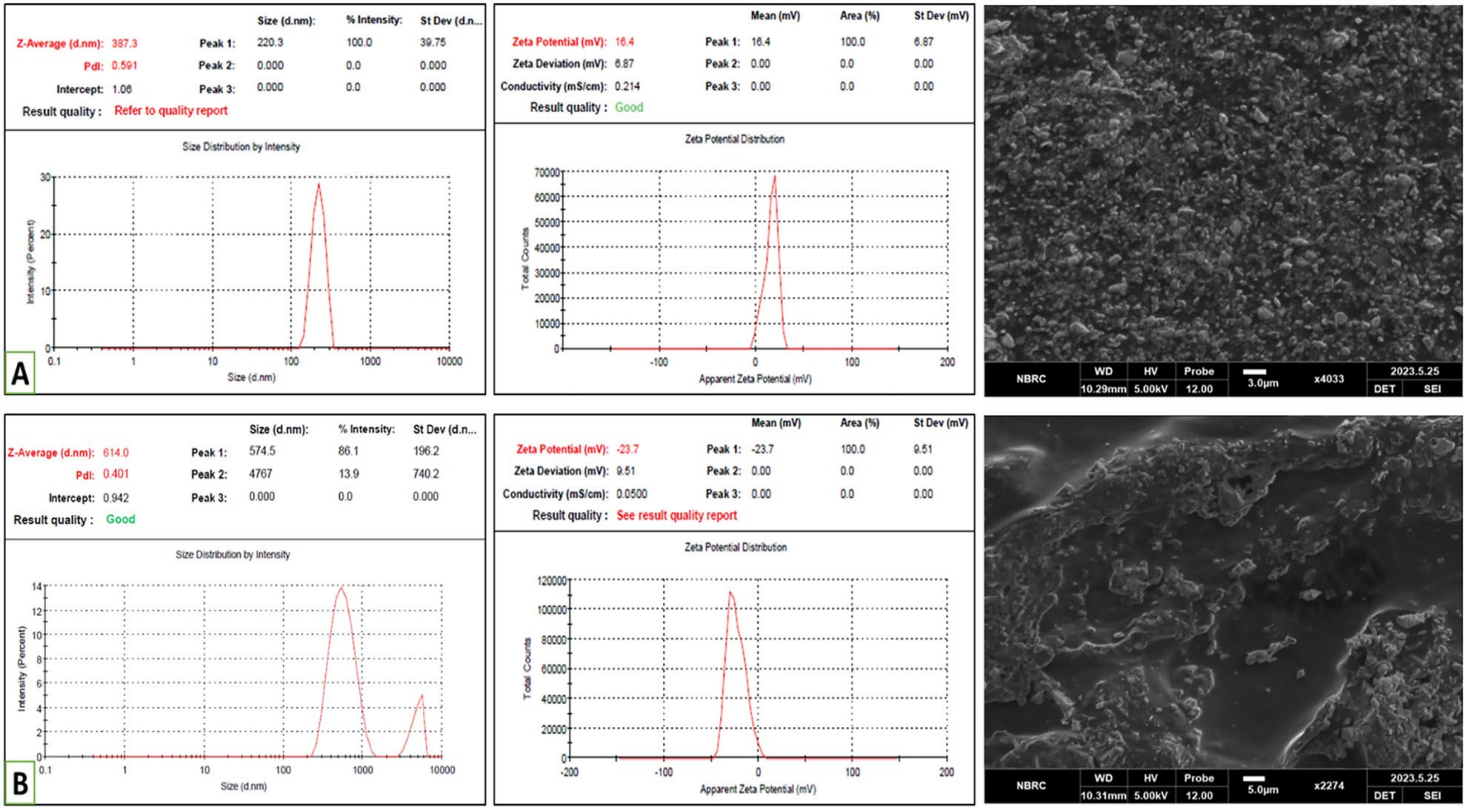
Reconstituted extemporaneous liquid formulation of HA-coated 5-FU in ThCs-NPs (**A**) particle size, PDI and zeta potential (left) and SEM analysis (right). Liquid formulation stored for 3 months (**B**) particle size, PDI and zeta potential (left) and SEM analysis (right) (mean + SD)^24,35,38^.

Furthermore, the legends of Figures 3, 5 and 6 contained errors.

Figure 3. Nanoparticle size (**A**), PDI (**B**), and Zeta potential (**C**) considerations in a Box-Behnken factorial design using DOE using results by dependent parameters to optimize nanoparticle formulation parameters.

now reads:

Figure 3: Nanoparticle size (**A**), PDI (**B**), and Zeta potential (**C**) considerations in a Box-Behnken factorial design using DOE using results by dependent parameters to optimize nanoparticle formulation parameters^24,35,38^.

Figure 5. SEM images (**A**: upper) show spherical and smooth surfaces of nanoparticles of Empty HA-ThCs and (**B**: upper) HA-coated 5-FU in ThCs-NPs at the scale of 5.0um while the TEM images (**A**: lower) showed more precise images of spherical and smooth surfaces of Empty HA-ThCs and (**B**: lower) HA-coated 5-FU in ThCs-NPs at scale of 50 nm and 200 nm^23,24,35^.

now reads:

Figure 5: SEM images (**A**: upper) show spherical and smooth surfaces of nanoparticles of Empty HA-ThCs and (**B**: upper) HA-coated 5-FU in ThCs-NPs at the scale of 5.0um while the TEM images (**C**) showed more precise image of HA-coated 5-FU in ThCs-NPs at scale of 50 nm^24,35,38^.

Figure 6. Functional group analysis of (**A**, a) Empty HA-ThCs and (**B**, b) HA-coated 5-FU in ThCs-NPs (Upper graphs for FTIR and lower graphs showed XRD analysis)^23,24,35^.

now reads:

Figure 6: Functional group analysis of (**A**, a) Empty HA-ThCs and (**B**, b) HA-coated 5-FU in ThCs-NPs (Upper graphs for FTIR and lower graphs showed XRD analysis)^24,35,38^.

The original Article has been corrected.

