# Supplementary material for: Enhancing therapeutic efficacy: sustained delivery of 5-fluorouracil (5-FU) via thiolated chitosan nanoparticles targeting CD44 in triple-negative breast cancer
Source: Sci Rep. 2024 May 19;14:11431. doi: 10.1038/s41598-024-55900-1 (PMC11102914; doi:10.1038/s41598-024-55900-1)
Supplement: Supplementary file 1 — Supplementary Tables. [file 41598_2024_55900_MOESM1_ESM.docx]

**Calibration Plot for 5-FU in Distilled Water and Phosphate Buffer at pH 7.4**

This procedure aimed to establish a calibration curve for 5-FU. Following the guidelines outlined in the British Pharmacopoeia (BP), 5-FU concentrations ranging from 0.1 to 1 mg/mL were prepared by diluting the drug in distilled water (served as a reference). The prepared samples were investigated using a UV-Vis spectrophotometer at 266 nm. Subsequently, a calibration curve was constructed utilizing MS-Excel, which played a pivotal role in determining unknown concentrations of 5-FU within samples of nanoparticle formulation [37].

A calibration curve based on drug concentration was constructed to measure the quantity of 5-FU in the release medium. Initially, 1 mg 5-FU was dissolved in 10 ml PBS (served as reference) at pH-7.4. The prepared solution was sonicated for 8 to 10 minutes to ensure thorough suspension. Then, the serial dilutions from this solution were prepared to plot the calibration curve using GraphPad Prism 9 software. The drug dilutions were analyzed at a wavelength of 266 nm using a UV spectrophotometer. The data was used to calculate the *in vitro* 5-FU release profile [38].

Table 1: Drug release rates from prepared nanoparticles compared to crude 5-FU at predefined time points in PBS at pH 7.4 and 6.8.

| **Sr. No.** | **Time (Hr.)** | **Absorbance**  **(pH 7.4)** | **Absorbance**  **(pH 6.8)** | **% 5-FU released from NPs at pH 7.4 (mean ± SD)** | **% 5-FU released from NPs at pH 6.8 (mean ± SD)** | **Crude 5-FU at pH 7.4 (mean ± SD)** |
| --- | --- | --- | --- | --- | --- | --- |
| 1 | 0 | 0 | 0 | 0 | 0 | 0 |
| 2 | 0.5 | 0.1123 | 0.1038 | 7.779119 | 26.75053467 | 7.779119206 |
| 3 | 1 | 0.1122 | 0.1129 | 15.54774 | 55.2900819 | 15.54774028 |
| 4 | 2 | 0.1101 | 0.1319 | 23.0959 | 62.20910204 | 23.09590048 |
| 5 | 4 | 0.1041 | 0.1418 | 30.01417 | 70.3048071 | 30.01417248 |
| 6 | 6 | 0.1038 | 0.1512 | 36.90095 | 77.7515013 | 36.90095008 |
| 7 | 12 | 0.1028 | 0.15156 | 43.68275 | 80.92601096 | 43.68274631 |
| 8 | 24 | 0.1109 | 0.15783 | 51.31489 | 81.63710112 | 51.31489161 |
| 9 | 48 | 0.1127 | 0.16128 | 66.15926 | 84.39398639 | 59.13600336 |
| 10 | 72 | 0.246 | 0.16907 | 80.19526 | 86.72478192 | 66.15925673 |

Table 2: Optimum kinetic model using cumulative percentages of 5-FU release in PBS (pH 7.4) at predefined intervals.

| **Sr. No.** | **Time (Hr.)** | **Cumulative % CsA released (pH 7.4)** | **Cumulative % CsA released (pH 6.8)** |
| --- | --- | --- | --- |
| 1 | 0 | 0 | 0 |
| 2 | 0.5 | 7.779119 | 26.75053467 |
| 3 | 1 | 15.54774 | 55.2900819 |
| 4 | 2 | 23.0959 | 62.20910204 |
| 6 | 4 | 30.01417 | 70.3048071 |
| 7 | 6 | 36.90095 | 77.7515013 |
| 8 | 12 | 43.68275 | 80.92601096 |
| 9 | 24 | 51.31489 | 81.63710112 |
| 10 | 48 | 66.15926 | 84.39398639 |
| 11 | 72 | 80.19526 | 86.72478192 |

Table 3: Kinetic models on 5-FU released from nanoparticles at pH mimics physiological and tumor microenvironment

|  | **Zero-order** | | **First-order** | | **Higuchi** | | **Korsmeyer-Peppas** | | **Hixon Crowell** | |
| --- | --- | --- | --- | --- | --- | --- | --- | --- | --- | --- |
|  | R^2^ | K_1_ | R^2^ | K_2_ | **R^2^** | **K_4_** | R^2^ | K_5_ | R^2^ | K_3_ |
| **pH 7.4** | 0.9389 | 7.137 | 0.9712 | -0.0413 | **0.9864** | **18.81** | 0.5902 | 10.263 | 0.6753 | 0.423 |
| **pH 6.8** | 0.6794 | 10.535 | 0.7212 | -0.0526 | **0.932** | **30.33** | 0.2554 | 0.7071 | 0.6579 | 0.1373 |
